# *N*‐acylhomoserine lactone‐regulation of genes mediating motility and pathogenicity in *Pseudomonas syringae* pathovar *tabaci* 11528

**DOI:** 10.1002/mbo3.440

**Published:** 2017-01-29

**Authors:** Feifei Cheng, Anzhou Ma, Jinxue Luo, Xuliang Zhuang, Guoqiang Zhuang

**Affiliations:** ^1^ Research Center for Eco‐Environment SciencesChinese Academy of SciencesBeijingChina; ^2^ University of the Chinese Academy of SciencesBeijingChina

**Keywords:** gene expression, *Pseudomonas syringae* pathovar *tabaci* 11528, quorum sensing, RNA sequencing, virulence traits

## Abstract

*Pseudomonas syringae* pathovar *tabaci* 11528 (*P. syringae* 11528) is a phytopathogen that causes wild‐fire disease in soybean and tobacco plants. It utilizes a cell density‐dependent regulation system known as quorum sensing (QS). In its QS system, the *psyI* is responsible for the biosynthesis of *N*‐acylhomoserine lactones (AHLs). By comparing the transcripts from *P. syringae* 11528 wild‐type strain with those of the Δ*psyI* mutant using RNA sequencing (RNA‐seq) technology, 1118 AHL‐regulated genes were identified in the transition from exponential to stationary growth phase. Numerous AHL‐regulated genes involved in pathogenicity were negatively controlled, including genes linked to flagella, chemotaxis, pilus, extracellular polysaccharides, secretion systems, and two‐component system. Moreover, gene ontology and pathway enrichment analysis revealed that the most pronounced regulation was associated with bacterial motility. Finally, phenotypic assays showed that QS‐regulated traits were involved in epiphytic growth of pathogens and disease development in plants. These findings imply that the AHL‐mediated QS system in *P. syringae* 11528 plays significant roles in distinct stages of interactions between plants and pathogens, including early plant colonization and late plant infection.

## 1 INTRODUCTION

Bacterial quorum sensing (QS), a cell–cell communication mechanism, is a gene regulation system that acts in a cell density‐dependent manner (Waters & Bassler, 2005). In QS system, signaling molecules, termed autoinducers, are produced and accumulated until they reach a critical threshold concentration, after which they bind to receptors that can trigger coordinated changes in gene expression (Keller & Surette, 2006). The most well‐known signaling molecules are *N*‐acylhomoserine lactones (AHLs) in Gram‐negative bacteria, which are mainly synthesized by LuxI‐homologs and are sensed by LuxR‐type regulator proteins, resulting in the subsequent regulation of bacterial gene expression and synergistic changes in physiological behaviors (Fuqua, Winans, & Greenberg, 1994). AHL‐mediated QS regulation plays a significant role in phytopathogens, as it is responsible for the expression of many traits involved in plant–pathogenic interactions (Loh, Pierson, Pierson, Stacey, & Chatterjee, 2002; Von Bodman, Bauer, & Coplin, 2003). The QS system in *Erwinia carotovora*, CarI/CarR, is required for the production of several exoenzymes involved in the maceration of plant tissues (Jones et al., 1993; Pirhonen, Flego, Heikinheimo, & Palva, 1993). In *Pantoea* (*Erwinia*) *stewartii*, exopolysaccharide as a virulence determinant is under the regulation of EsaI/EsaR system (Von Bodman, Majerczak, & Coplin, 1998). *P. aeruginosa*, a pathogen of both plants and animals, has two separate but interrelated QS systems, and the LasI/LasR system controls the expression of the RhlI/RhlR system (Schuster and Greenberg, 2006). Both QS systems regulate a large amount of extracellular virulence factors and secondary metabolites, among which some factors contribute to the epiphytic growth of bacteria in planta (Heurlier, Denervaud, & Haas, 2006; Rahme et al., 2000; Smith, 2003; Whitehead, Barnard, Slater, Simpson, & Salmond, 2001).

The pathogenicity of phytopathogens depends upon various factors, including effectors secreted by a type III secretion system (TTEs), toxins, and epiphytic fitness (Baltrus et al., 2011; Studholme et al., 2009). Among these factors, epiphytic fitness, which represents the ability to acquire nutrients and survive on the surface of a leaf (Lindeberg, Myers, Collmer, & Schneider, 2008), is influenced by many determinants, such as motility (Quiñones, Dulla, & Lindow, 2005), chemotaxis (Brencic & Winans, 2005; Yao & Allen, 2006), extracellular polysaccharides (EPS) (Taguchi et al., 2008; Von Bodman & Farrand, 1995; Von Bodman et al., 1998), and iron uptake (Cha, Lee, Oh, Choi, & Baik, 2008). Moreover, cell wall‐degrading hydrolytic enzymes (Mäe, Montesano, Koiv, & Palva, 2001) and other secretion systems (Jakob, Kniskern, & Bergelson, 2007; Preston, Studholme, & Caldelari, 2005) may also contribute to both virulence and epiphytic fitness. Multidrug resistance mediated by multidrug efflux pump, is involved in the resistance to antimicrobials produced by plants and contributes to bacterial virulence (Barabote et al., 2003; Burse, Weingart, & Ullrich, 2004). Many studies have reported that the expression of many genes involved in pathogenicity is regulated by QS in phytopathogens, including *Pantoea stewartii* (Von Bodman & Farrand, 1995), *Ralstonia solanacearum* (Clough, Flavier, Schell, & Denny, 1997), *Erwinia carotovora* (Mäe et al., 2001), and *Burkholderia glumae* PG1 (Gao et al., 2015).

Quorum sensing regulation is also very common to *Pseudomonas syringae*, a widespread bacterial pathogen that causes disease in a broad range of economically important plant species. The production of AHL effects colony morphology as well as epiphytic viability in *P. syringae* pv. *syringae* strain B3A (Dumenyo, Mukherjee, Chun, & Chatterjee, 1998). In addition to epiphytic fitness, EPS and motility are also regulated by AhlI/AhlR system of *P. syringae* pv. *syringae* B728a (Quiñones et al., 2005). *P. syringae* pv. *tabaci*, one of *P. syringae* pathovars, causes wild‐fire disease in soybeans and tobacco plants (Gasson, 1980; Ribeiro, Hagedorn, Durbin, & Uchytil, 1979). *P. syringae* pv. *tabaci* synthesizes a phytotoxin, termed tabtoxin, which generates a hydrolytic product tabtoxinine‐β‐lactam that can inhibit glutamine synthetase and contributes to the accumulation of ammonia and subsequent loss of chlorophyll in the host cell, ultimately resulting in the formation of chlorotic halos that surround necrotic spots on the leaves of infected plants (Durbin, 1991; Thomas, Langstonunkefer, Uchytil, & Durbin, 1983). The QS system of *P. syringae* pv. *tabaci* depends on *psyI*, which is responsible for the biosynthesis of AHLs, as well as the regulatory protein PsyR (Elasri et al., 2001). The production of *N*‐(3‐oxo‐hexanoyl)‐homoserine lactone (3OC6‐HSL) and *N*‐hexanoyl‐L‐HSL (C6‐HSL) has been reported in *P. syringae* pv. *tabaci* (Shaw et al., 1997; Taguchi et al., 2006). Several phenotypic assays have been reported that the *psyI‐*deficiency could affect some bacterial behaviors including a few virulence traits in *P. syringae* pv. *tabaci* (Taguchi et al., 2006). But limited further analysis is available concerning the role of AHL‐mediated QS regulation on the pathogenicity of *P. syringae* pv. *tabaci*. Notably, we do not know the number and types of virulence traits under the regulation of QS system, and how AHL controls virulence traits has not been studied systematically. Thus, the comprehensive exploration of QS‐dependent regulons is urgently needed in *P. syringae* pv. *tabaci*.

In this study, we investigated the AHL‐mediated QS regulation of *P. syringae* pv. *tabaci* 11528 (*P. syringae* 11528), which naturally causes disease in wild tobacco, an important model system for studying plant–pathogen interactions, and its draft complete genome sequence has been analyzed (Studholme et al., 2009). We compared the transcripts from the Δ*psyI* mutant with those of the wild type during growth using RNA sequencing (RNA‐seq) technology. Phenotypic assays designed to assess plant–pathogen interactions, including swarming motility and disease symptoms, were comparatively analyzed. These findings will extend our understanding of AHL‐mediated regulation on plant–pathogen interactions and provide the molecular basis for the pathogenicity in *P. syringae* pv. *tabaci*.

## 2 MATERIALS AND METHODS

### 2.1 Bacterial strains, plasmids, and culture conditions

The bacterial strains and plasmids used in this study are listed in Table**** 1. *Agrobacterium tumefaciens* A136 and *Chromobacterium violaceum* CV026 were cultivated at 30°C in LB broth (McLean, Whiteley, Stickler, & Fuqua, 1997). *P. syringae* pv. *tabaci* 11528 (*P. syringae* 11528) and the Δ*psyI* mutant were grown at 30°C in King's medium B (KB) (Cha et al., 2008). *Escherichia coli* DH5α was grown at 37°C in LB broth. Antibiotics were added to media as required at the following final concentrations: ampicillin, 50 μg/μl; spectinomycin, 50 μg/μl; tetracycline, 20 μg/μl; and kanamycin, 20 μg/μl.

**Table 1:** Bacterial strains and plasmids used in this study

Strains or plasmids	Relevant characteristics^a^	Source or reference
Strains		
*P. syringae* pv. *tabaci* 11528	Tox^+^ Tox^r^, causal agent of wild‐fire of tobacco (ATCC type strain)	ATCC, USA
*P. syringae* pv. *tabaci* △*psyI* mutant	ATCC 11528 △*psyI* (683‐bp deletion)	Cha et al. (2008)
*A. tumefaciens* A136	Tet^r^,Sp^r^,*traI‐lacZ* fusion; AHL biosensor	McLean et al. (1997)
*C. violaceum* CV026	Km^r^,AHL biosensor	McLean et al. (1997)
*Escherichia coli* DH5α	F^‐^φDH5lacZ ΔM15 Δ(*lacZYA‐argF*)U169 end A1 recA1 hsdR17(r_k_^‐^m_k_^‐^) supE44 λ‐ thi‐1 gyrA96 relA1 phoA	Biomed, Peking, China
Pta(pBQ9)	Sp^r^ *, P. syringae* pv. tabaci 11528 wild type containing plasmid pBQ9	Cheng et al.(2016)
△*psyI*(pBQ9‐P_nptII_)	Sp^r^ *, P. syringae* pv. tabaci 11528 △*psyI* mutant containing plasmid pBQ9‐P_nptII_	This study
Plasmids		
pBQ9	Sp^r^, pPROBE‐OT derivative harboring *P. syringae ahlI* promoter upstream of GFP	Quiñones et al. (2005)
pUCGMA2T_1−4_	Amp^r^, *P*_*nptII*_:gfp	Deng et al. (2014)
pBQ9‐P_nptII_	Sp^r^, derivative of pBQ9 containing *P*_*nptII*_ promoter	This study

Tox^r^, tabtoxin resistance; Tet^r^, tetracycline resistance; Km^r^, kanamycin resistance; Sp^r^, spectinomycin resistance; Amp^r^, ampicillin resistance.

### 2.2 RNA isolation

To prepare RNA samples, *P. syringae* 11528 wild‐type strain and Δ*psyI* mutant were grown in a shaker incubator at 30°C and 200 rpm to exponential phase after inoculated into test tubes with 5 ml KB and then subcultured in conical flasks with 50 ml KB. Cells were harvested at lag phase (L phase; OD_600_ of 0.3), exponential phase (E phase; OD_600_ of 0.7) and a transition from exponential to stationary phase (T phase; OD_600_ of 1.7) by centrifugation at 5,000***g*** for 5 min. Total RNA was extracted from ~10 mg of cell pellets using a miRNeasy Mini Kit (Qiagen, Hilden, Germany) according to the previous study (Cheng, Ma, Zhuang, & Fray, 2016). Three biological replicates were processed per sample.

### 2.3 RNA‐seq library construction and sequencing

RNA‐seq libraries were generated using the NEBNext^®^ Ultra^™^ Directional RNA Library Prep Kit for Illumina^®^ (New England BioLabs, Inc., Ipswich, MA, USA) following the manufacturer's recommendations. One hundred base‐pair, paired‐end sequencing was performed using an Illumina HiSeq 2000 platform. Raw reads were subjected to standard quality control criteria to remove all reads that fit any of the following parameters: reads that aligned to adaptor sequences; reads for which more than 10% of bases were unknown; reads for which there were more than 50% of low‐quality bases (quality value <5) in one read. All remaining clean reads were mapped to *P. syringae* 11528 genome (Studholme et al., 2009) (sequence download from http://www.ncbi.nlm.nih.gov/Traces/wgs/?val=ACHU02#contigs) and analyzed using Bowtie2‐2.0.6 (Langmead & Salzberg, 2012). Those reads that mapped to the reference genome were used in further analyses. Sequence‐read mapping and genome coverage data are summarized in Table**** 2. The number of reads that mapped to each gene was counted using HTSeq v0.6.1. Levels of gene expression were calculated using the RPKM (reads per kilobase transcriptome per million reads) method (Mortazavi, Williams, McCue, Schaeffer, & Wold, 2008).

**Table 2:** Overall statistics of RNA‐seq data

Growth phase^a^	Sample name^b^	Clean reads	Uniquely mapped	Mapping ratio
L phase	WT (1)	8,374,920	7,960,118	95.05%
	WT (2)	9,020,734	8,581,603	95.05%
	WT (3)	12,223,654	11,596,127	94.87%
	MT (1)	11,451,222	10,859,077	94.83%
	MT (2)	11,384,438	10,822,555	95.06%
	MT (3)	10,778,120	10,245,952	95.06%
E phase	WT (1)	10,536,414	10,011,486	95.02%
	WT (2)	9,466,758	8,962,413	94.67%
	WT (3)	10,374,856	9,782,692	94.29%
	MT (1)	8,628,616	8,261,892	95.75%
	MT (2)	13,608,578	12,996,703	95.5%
	MT (3)	13,823,208	13,219,185	95.63%
T phase	WT (1)	8,228,786	7,913,448	96.17%
	WT (2)	10,591,606	10,206,726	96.37%
	WT (3)	9,670,798	9,205,368	95.19%
	MT (1)	12,170,034	11,523,821	94.69%
	MT (2)	33,263,468	31,646,901	95.14%
	MT (3)	23,287,204	22,186,569	95.27%

a Lag phase (L phase); Exponential phase (E phase); Transition from exponential to stationary phase (T phase).

b Wild‐type strain (WT); Δ*psyI* mutant (MT). Replicate numbers are indicated in parentheses.

### 2.4 RNA‐seq analysis

Differential expression analysis data were displayed using the DESeq R package (1.10.1) (Anders & Huber, 2010). All resulting *p*‐values were adjusted using the Benjamini–Hochberg procedure to control the false discovery rate (FDR). Those genes with an adjusted *p*‐value <.05 and at least a twofold change in expression were classified as differentially expressed genes (DEGs). To analyze DEGs, we conducted a gene ontology (GO; http://www.geneontology.org/) enrichment assay using the GOseq R package (Young, Wakefield, Smyth, & Oshlack, 2010) and then calculated an adjusted *p*‐value. An adjusted *p*‐value <.05 was considered to indicate significance for the enriched set of data. For subsequent analyses of the QS‐dependent pathway, DEGs were mapped to the pathway database (http://www.genome.jp/kegg/) using KOBAS 2.0 software (Mao, Tao, & Wei, 2005). The mapped pathway was enriched using an adjusted *p*‐value of <.05.

### 2.5 Quantitative real‐time PCR

To validate the gene expression changes indicated by the RNA‐seq data, quantitative real‐time PCR (qPCR) analysis was performed according to the protocol of Cheng et al. (2016). Specific primers for qPCR are listed in Table S1. Quantification of transcript expression was carried out using the 2^−ΔΔCt^ method using the constitutively expressed 16S ribosomal RNA gene as a control for normalization.

### 2.6 Swarming motility tests

Swarming motility was assessed on semisolid KB plates that contained 0.4% (wt/vol) Bacto agar (BD‐Difco) according to a previous study (Taguchi et al., 2006) with some modifications. Cells of *P. syringae* 11528 wild‐type strain and Δ*psyI* mutant were grown to T phase and then resuspended in 10 mmol/L potassium phosphate buffer (PBS). Two microliter of suspensions (~10^7^ cells) was dropped onto KB plates and then inoculated plates were incubated at 27°C for 36 hr, and colony diameters were measured as indicators of swarming motility. All experiments were conducted with three replicates and repeated three times.

### 2.7 GFP‐labeling of *P. syringae* 11528 Δ*psyI* mutant

GFP‐labeled *P. syringae* 11528 wild‐type strain, Pta(pBQ9), and its observation on tobacco leaves were reported in our previous works (Cheng et al., 2016). The plasmid pBQ9 encodes a *gfp* marker gene that is fused to the promoter of *P*_*ahlI*_ and yields inducible expression of green fluorescence in *P. syringae* (Quiñones, Pujol, & Lindow, 2004). The promoter for *P*_*nptII*_ is a neomycin phosphotransferase promoter that constitutively expresses the *gfp* gene (Stiner & Halverson, 2002). For GFP‐labeling of *P. syringae* 11528 Δ*psyI* mutant, we constructed a plasmid pBQ9‐P_nptII_, which yields constitutive expression of green fluorescence. A fragment of the promoter *P*_*nptII*_ was amplified from plasmid pUCGMA2T_1‐4_ (Deng, Zhuang, Ma, Yu, & Zhuang, 2014) using primers nptII‐1 (CCC*GTCGAC*GTCAGGCTGTAACAGCTCAGA) and nptII‐2 (GGC*GAATTC*ATCCTGTCTCTTGATCAGATCTTG) that contained engineered *Sal*I and *Eco*RI restriction enzyme sites (italicized with underline), and the fragment was then cloned into plasmid pBQ9 as a *Sal*I‐*Eco*RI fragment at the 5ʹ‐terminus of the *gfp* gene, replacing the entire *P*_*ahlI*_ promoter and creating plasmid pBQ9‐P_nptII_. Δ*psyI*(pBQ9‐P_nptII_) was constructed by electroporation of the pBQ9‐P_nptII_ plasmid into the Δ*psyI* mutant. Thus, GFP‐labeled Δ*psyI* mutant was achieved.

### 2.8 Epifluorescence microscopy on tobacco surfaces

To study the ability of both *P. syringae* 11528 wild‐type strain and Δ*psyI* mutant to colonize plants, tobacco leaves (*Nicotiana tabacum* L.) were spray‐inoculated to wetness with a suspension that contained 10^6 ^CFU/ml of GFP‐labeled *P. syringae* 11528 wild‐type or Δ*psyI* mutant cells. After inoculation, tobacco plants were placed in a greenhouse (25°C, ~80% relative humidity, 12 hr/day photoperiod). GFP‐labeled *P. syringae* 11528 strains were observed using a confocal laser scanning microscope (CLSM; LSM 780, Carl Zeiss, Oberkochen, Germany) at 1 and 3 days following inoculation and bacterial population was determined following Cheng et al. (2016).

### 2.9 Pathogenicity tests in tobacco leaves

The pathogenicity of both *P. syringae* 11528 wild‐type strain and Δ*psyI* mutant was evaluated in tobacco plants. Bacterial cells were grown to T phase and 10 μl suspensions of ~10^8^ CFU/ml were infiltrated into tobacco leaves according to a previously published protocol (Cha et al., 2008). Inoculated tobacco plants were incubated in a greenhouse. Plants were evaluated for disease symptoms and diameters of the necrotic lesions that formed at each inoculation site were measured as indicators of virulence at 1, 3, 5, 7, and 9 days after inoculation. For each treatment, 20 leaves with two inoculation sites per leaf were randomly selected.

### 2.10 Statistical analysis

The results of bacterial swarming motility, epiphytic population size, and lesion size on tobacco leaves are expressed as the means ± Standard Error of Mean. One‐way analysis of variance (ANOVA) and *t* test were performed using software Graphpad Prism V6.0 (GraphPad Software, San Diego, CA) for data analysis. *^*^ *p *<* *.01; *^**^ *p *<* *.001; *^***^ *p *<* *.0001.

### 2.11 SRA accession number

The clean reads of RNA‐seq have been deposited in the NCBI sequence‐read archive (SRA) database under accession no. SRP078136.

## 3 RESULTS

### 3.1 Overview of RNA‐seq data

Before RNA‐seq, we assayed the AHL‐producing potential of *P. syringae* 11528 Δ*psyI* mutant using AHL‐biosensor strains *C. violaceum* CV026 and *A. tumefaciens* A136 (McLean et al., 1997; Steindler & Venturi, 2007) according to the previous studies (Lv et al., 2013). The Δ*psyI* mutant did not induce the production of pigments in AHL‐biosensor strains (Figure S1), indicating the absence of bioactive AHL. These data imply that the Δ*psyI* mutant was extremely defective in AHL production. Moreover, transcriptome analysis established that the *psyI* was only transcribed in *P. syringae* 11528 wild‐type strain, as no *psyI* mRNA could be detected in *P. syringae* 11528 Δ*psyI* mutant (Gene locus: PSYTB_23601, Table S2). Thus, we suggest that the *psyI* in *P. syringae* 11528 is the only gene that encodes AHL synthases.

To characterize the regulatory function of the AHL‐mediated QS system in *P. syringae* 11528, RNA‐seq analysis was conducted for *P. syringae* 11528 wild‐type strain and Δ*psyI* mutant. We identified transcripts of each sample with three biological replicates in the lag (L), exponential (E), and transition (T) phases, and the resulting 18 libraries yielded 8,228,786 to 33,263,468 clean reads (Table 2). To profile the gene expression patterns, we mapped the clean reads of the 18 libraries to *P. syringae* 11528 genome. Overall, 7,913,448 to 31,646,901 clean reads were uniquely mapped and the frequency of mapped reads was 94.29–96.37% (Table 2), indicating that effective reads of RNA‐seq data were well mapped to the reference genome. To validate the RNA‐seq data, qPCR was conducted for expression analysis using 22 randomly selected genes (Table S1), and the relative expression levels of these genes between the Δ*psyI* mutant and the wild type were compared. Data from qPCR largely confirmed the RNA‐seq data, and a positive correlation was detected between them (Figure S2), suggesting that RNA‐seq data were robust and of good quality.

### 3.2 QS‐dependent gene expression patterns in *P. syringae* 11528

To identify AHL‐regulated genes in *P. syringae* 11528, we compared the transcripts of *P. syringae* 11528 wild‐type strain with those of the Δ*psyI* mutant. As shown in Table 3, 31 and 41 differentially expressed genes (DEGs) were QS‐dependent regulated in the L and E phases, respectively, while a total of 1118 genes were found to be differentially expressed in the T phase. These data indicated that few genes were regulated during early growth, including L and E phases, and most genes were not controlled by QS until T phase. Thus, AHL‐dependent QS regulation was the most significant during T phase, and we focused on the T‐phase RNA‐seq analysis in subsequent assessments.

**Table 3:** Numbers of QS‐regulated genes in *Pseudomonas syringae* pv. *tabaci* 11528

Growth phase^a^	No. of differentially expressed genes (DEGs)		
	Up‐regulated	Down‐regulated	Total
L phase	30	1	31
E phase	38	3	41
T phase	407	711	1118

a Lag phase (L phase); Exponential phase (E phase); Transition from exponential to stationary phase (T phase).

Among AHL‐regulated genes, many genes were associated with epiphytic fitness or virulence on plants (Table 4). Regarding bacterial pilus, *P. syringae* 11528 Δ*psyI* mutant showed enhanced expression of genes that encode pilus assembly proteins. For secretion systems, type II, type III, and type VI secretion systems were down‐regulated in a QS‐dependent manner, with the exception of several individual genes. Regarding the two‐component system, six related genes were found to be down‐regulated. In addition, multidrug efflux pump and EPS genes were mostly down‐regulated by QS. Three genes related to iron transport were positively regulated by AHL, as was PSYTB_09751 that encodes a binary cytotoxin component, as shown in Table**** 4. The most dramatic effect of QS regulation on gene expression was observed for genes linked to flagella and chemotaxis. Thirty‐eight and 45 genes responsible for the synthesis of bacterial flagella and chemotaxis proteins, respectively, were completely down‐regulated by AHL (Table**** 4). These data suggest that AHL‐dependent QS regulation in *P. syringae* 11528 negatively regulates the expression of a variety of virulence traits, and presumably plays a significant role in plant–pathogen interactions.

**Table 4:** QS‐regulated genes related to virulence traits in *Pseudomonas syringae* pv. *tabaci* 11528

Virulence traits	Gene locus^a^	Predicted function	Fold change^b^
Flagella	PSYTB_08676	Flagellar motor protein	−3.28
	PSYTB_08681	Flagellar motor protein	−3.54
	PSYTB_15355	Flagellar synthesis chaperone protein	−2.66
	PSYTB_15365	Flagellar basal body P‐ring biosynthesis protein	−6.34
	PSYTB_15395	Flagellar biosynthesis protein	−5.37
	PSYTB_15400	Flagellar basal body rod protein	−5.83
	PSYTB_15405	Flagellar basal body rod modification protein	−6.33
	PSYTB_15410	Flagellar hook protein	−5.56
	PSYTB_15415	Flagellar hook protein	−3.18
	PSYTB_15420	Flagellar basal body rod protein	−9.29
	PSYTB_15425	Flagellar basal body rod protein	−10.23
	PSYTB_15430	Flagellar L‐ring protein	−10.56
	PSYTB_15435	Flagellar P‐ring protein	−11.43
	PSYTB_15440	Flagellar rod assembly protein	−11.26
	PSYTB_15445	Flagellar hook protein	−5.34
	PSYTB_15455	Flagellar hook‐associated protein	−4.07
	PSYTB_15485	Flagellar protein	−2.40
	PSYTB_15495	Flagellar protein	−4.14
	PSYTB_15500	Flagellar assembly protein	−4.45
	PSYTB_15520	Flagellar hook‐basal body protein	−8.37
	PSYTB_15525	Flagellar M‐ring protein	−10.06
	PSYTB_15535	Flagellar motor switch protein	−9.86
	PSYTB_15540	Flagellar assembly protein	−7.49
	PSYTB_15550	Flagellar protein	−4.18
	PSYTB_15570	Flagellar hook‐length control protein	−4.52
	PSYTB_15575	Flagellar basal body‐associated protein	−5.69
	PSYTB_15580	Flagellar motor switch protein	−9.96
	PSYTB_15585	Flagellar motor switch protein	−9.88
	PSYTB_15590	Flagellar assembly protein	−8.39
	PSYTB_15595	Flagellar biosynthesis protein	−7.68
	PSYTB_15600	Flagellar biosynthetic protein	−8.81
	PSYTB_15605	Flagellar biosynthesis protein	−4.93
	PSYTB_15610	Flagellar biosynthesis protein	−4.77
	PSYTB_15615	Flagellar biosynthesis protein	−7.51
	PSYTB_15620	Flagellar biosynthesis regulator	−9.15
	PSYTB_15670	Flagellar motor protein	−3.58
	PSYTB_15665	Flagellar motor protein	−3.62
	PSYTB_15885	Flagellar hook‐length control protein	−2.74
Chemotaxis	PSYTB_00315	Methyl‐accepting chemotaxis protein	−3.45
	PSYTB_01319	Methyl‐accepting chemotaxis protein	−4.21
	PSYTB_01504	Chemotaxis protein	−4.24
	PSYTB_03144	Chemotaxis protein	−6.97
	PSYTB_03626	Chemotaxis protein	−3.02
	PSYTB_03656	Chemotaxis protein	−3.64
	PSYTB_03756	Chemotaxis protein	−2.51
	PSYTB_03841	Methyl‐accepting chemotaxis protein	−4.15
	PSYTB_05055	Chemotaxis protein	−9.00
	PSYTB_05130	Chemotaxis protein	−4.48
	PSYTB_06027	Chemotaxis protein	−2.48
	PSYTB_06032	Chemotaxis protein	−2.52
	PSYTB_12048	Chemotaxis protein	−3.22
	PSYTB_12073	Methyl‐accepting chemotaxis protein	−2.27
	PSYTB_12233	Chemotaxis protein	−2.44
	PSYTB_12238	Chemotaxis protein	−2.38
	PSYTB_13085	Chemotaxis protein	−5.00
	PSYTB_14413	Chemotaxis protein	−2.37
	PSYTB_15370	Chemotaxis protein	−3.51
	PSYTB_15375	Chemotaxis protein	−3.05
	PSYTB_15640	Chemotaxis protein	−4.00
	PSYTB_15650	Chemotaxis protein	−3.32
	PSYTB_15680	Chemotaxis protein	−3.63
	PSYTB_15685	Chemotaxis protein	−4.02
	PSYTB_15690	Chemotaxis protein	−3.43
	PSYTB_15700	Chemotaxis protein	−3.24
	PSYTB_15805	Chemotaxis protein	−2.36
	PSYTB_16125	Chemotaxis protein	−3.39
	PSYTB_16140	Methyl‐accepting chemotaxis protein	−3.09
	PSYTB_16410	Chemotaxis protein	−3.33
	PSYTB_16590	Methyl‐accepting chemotaxis protein	−0.37
	PSYTB_17560	Methyl‐accepting chemotaxis protein	−2.04
	PSYTB_19011	Methyl‐accepting chemotaxis protein	−2.99
	PSYTB_20081	Chemotaxis protein	−3.53
	PSYTB_20086	Methyl‐accepting chemotaxis protein	−3.92
	PSYTB_24172	Chemotaxis protein	−2.55
	PSYTB_25716	Chemotaxis protein	−4.77
	PSYTB_25726	Chemotaxis protein	−4.58
	PSYTB_25736	Chemotaxis protein	−4.55
	PSYTB_25746	Chemotaxis protein	−3.21
	PSYTB_25761	Chemotaxis protein	−2.32
	PSYTB_27487	Chemotaxis protein	−10.16
	PSYTB_27757	Chemotaxis protein	−4.70
	PSYTB_28392	Chemotaxis sensory transducer	−3.26
Pilus	PSYTB_07811	Pilus assembly protein	−2.45
	PSYTB_07816	Pilus assembly protein	−2.13
	PSYTB_07821	Pilus assembly protein	−2.21
	PSYTB_09526	Pilus assembly protein	−3.32
	PSYTB_15350	Pilus assembly protein	−2.25
	PSYTB_23496	Pilus assembly protein	−2.32
Secretion system	PSYTB_01314	Type VI secretion protein	−2.80
	PSYTB_03239	Type VI secretion protein	4.84
	PSYTB_03249	Type VI secretion protein	2.69
	PSYTB_14001	Type II secretion system protein	−3.09
	PSYTB_14013	Type II secretion system protein	−3.56
	PSYTB_14018	Type II secretion system protein	−3.60
	PSYTB_21360	Type VI secretion protein	−5.51
	PSYTB_21365	Type VI secretion protein	−5.02
	PSYTB_21370	Type VI secretion protein	−5.05
	PSYTB_21375	Type VI secretion system protein	−4.11
	PSYTB_21380	Type VI secretion system protein	−3.41
	PSYTB_21410	Type VI secretion system protein	−2.01
	PSYTB_21420	Type VI secretion system effector	−2.38
	PSYTB_21425	Type VI secretion protein	−2.47
	PSYTB_23396	Type II secretion system protein	2.08
	PSYTB_27882	Type III effector	−2.07
Two‐component system	PSYTB_04915	Two‐component system response regulator	−2.96
	PSYTB_05410	Two‐component system sensor histidine kinase	−2.97
	PSYTB_17485	Two‐component system response regulator	−3.52
	PSYTB_22030	Two‐component sensor histidine kinase	−2.10
	PSYTB_25756	Two‐component system response regulator	−3.47
	PSYTB_26762	Two‐component system response regulator	−4.47
Iron transport	PSYTB_09216	Iron dicitrate transporter	3.94
	PSYTB_09221	Iron ABC transporter	2.85
	PSYTB_09226	Iron siderophore‐binding protein	3.81
Multidrug efflux pump	PSYTB_06721	Multidrug ABC transporter permease	2.20
	PSYTB_26650	Multidrug ABC transporter substrate	−2.10
	PSYTB_04905	Multidrug transporter	−2.32
Extracellular polysaccharide	PSYTB_04635	Alginate biosynthesis protein	−2.46
	PSYTB_15990	Alginate lyase	−4.65
Toxin	PSYTB_09751	Binary cytotoxin component	2.97

a Gene locus corresponds to the *P. syringae* pv. *tabaci* 11528 genome.

b Fold change of gene expression in the wild type in comparison of the Δ*psyI* mutant; The minus sign before fold change corresponds to down‐regulation by AHL‐mediated QS system.

**Figure 1:**
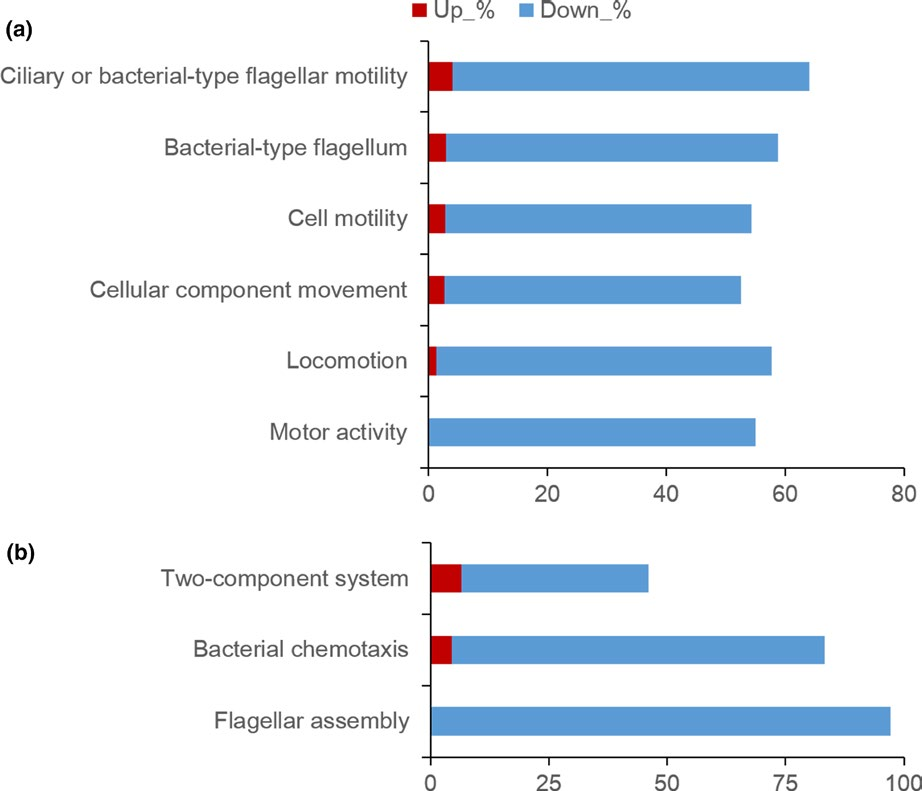
Analysis of Quorum sensing (QS)‐dependent genes. QS‐dependent genes were identified by comparing transcriptomes of *Pseudomonas syringae* 11528 wild‐type strain with those of the Δ*psyI* mutant. Gene ontology (GO) enrichment terms of QS‐dependent genes linked to bacterial motility (a); Enrichment pathways of QS‐dependent genes (b). Each bar represents the percentage of genes (red, up‐regulated; blue, down‐regulated) belonging to the GO term or pathway shown in the *y*‐axis

**Figure 2:**
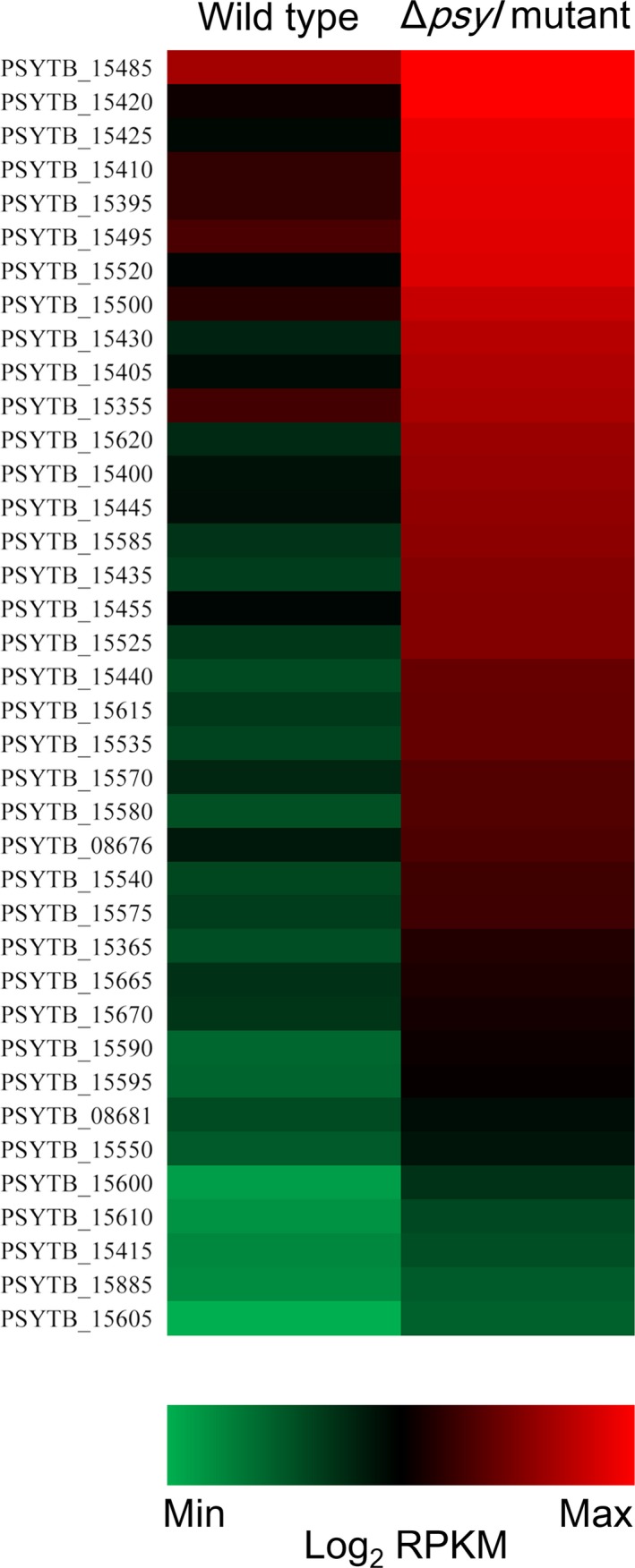
Expression profiles of genes linked to flagellar assembly. Each column of the heat map represents the Log_2_ RPKM of each gene in *Pseudomonas syringae* 11528 wild‐type strain (left) and the Δ*psyI* mutant (right) with a green‐black‐red scheme. Red, high expression; green, low expression

To identify specific processes that were more prominently AHL‐dependent in *P. syringae* 11528, AHL‐regulated genes were grouped into functional categories by GO and pathway enrichment analysis. Overall, a set of 1118 AHL‐regulated genes were assigned to 1054 GO biological process terms and enriched in 41 terms, 210 GO cellular component terms and enriched in 11 terms, and 558 GO molecular function terms and enriched in three terms. Among those enrichment terms, many terms were closely related to bacterial motility, such as locomotion, cell motility, cellular component movement, and motor activity (Figure 1a). The maximal ratio of up‐regulation of those terms was only 4%, while motor activity was completely repressed by AHL‐mediated QS system. The distribution of enrichment pathways showed that three pathways were enriched, including two‐component system, bacterial chemotaxis, and flagellar assembly; the two latter pathways were involved in bacterial motility, of which flagellar assembly was completely down‐regulated in an AHL‐dependent manner (Figure 1b). To represent the effect of QS on flagella in a different format, expression profiles of genes linked to flagellar assembly were comparatively assessed using a heat‐map analysis (Figure 2), which revealed a significantly higher expression of flagellum protein in *P. syringae* 11528 Δ*psyI* mutant than that in the wild type.

### 3.3 Phenotypic analyses of *P. syringae* 11528 wild type and Δ*psyI* mutant

#### 3.3.1 Bacterial motility

**Figure 3:**
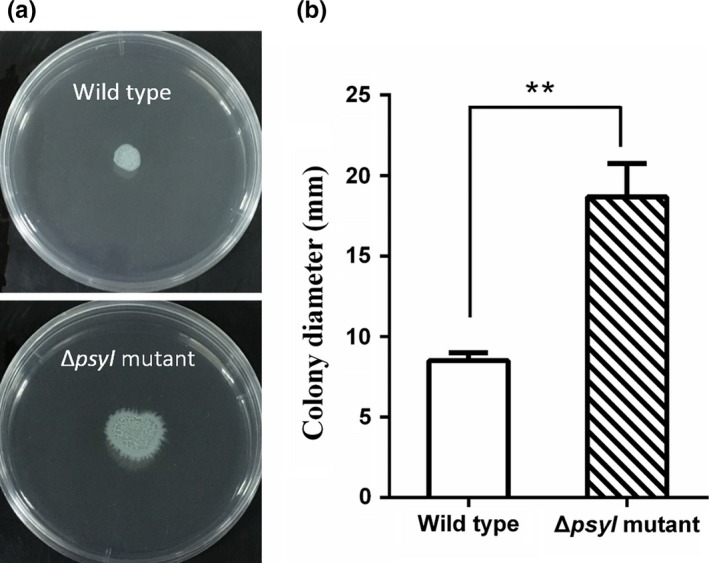
Quorum sensing (QS)‐dependent swarming motility. Swarming motility phenotype (a) and swarming distance (b) of *Pseudomonas syringae* 11528 wild‐type strain (top) and the Δ*psyI* mutant (bottom) on semisolid King's B (KB) plates. Sterile filter discs placed on KB semisolid plates were inoculated with 1 × 10^7^ cells and plates were incubated at 27°C for 36 hr

When cells of *P. syringae* 11528 wild type and Δ*psyI* mutant were inoculated on low‐agar plates at 27°C for 36 hr, the Δ*psyI* mutant showed irregular dendritic colony pattern, which is a typical characteristic of *P. syringae* AHL‐mutant swarming behavior (Quiñones et al., 2005; Taguchi et al., 2006), while it was not observed on the plates of the wild type (Figure 3a). Moreover, the Δ*psyI* mutant cells spreaded rapidly away from the point of inoculation but the wild‐type cells exhibited limited swarming motility. We measured colony diameter to indicate swarming motility. Colony diameters of the Δ*psyI* mutant was ~2.2‐fold larger than that of the wild type (Figure 3b). Results imply that swarming motility is repressed by AHL‐mediated QS system in *P. syringae* 11528.

#### 3.3.2 Colonization observation

**Figure 4:**
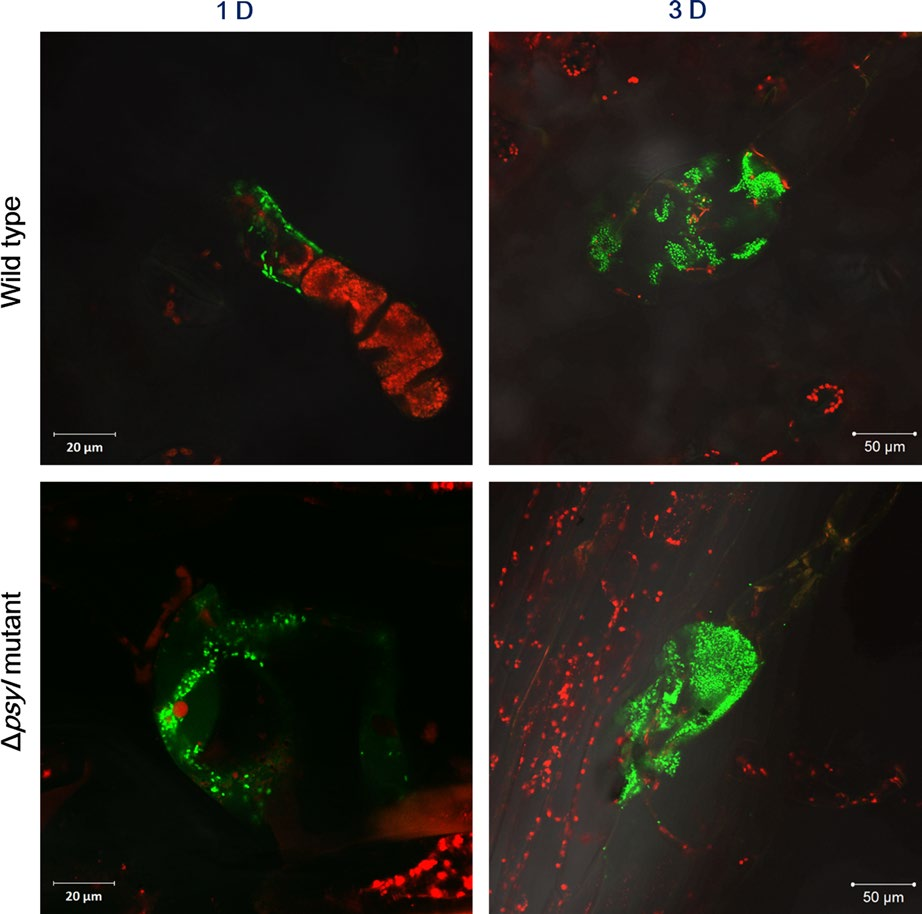
Colonization of GFP‐labeled *Pseudomonas syringae* 11528 strains on tobacco leaves. Tobacco leaves were spray‐inoculated with P. syringae pv. tabaci 11528 wild‐type strain (top) or the Δ*psyI* mutant (bottom) at the concentrations of 10^6 ^CFU/ml and were observed in the glandular trichomes at 1 and 3 days after inoculation by confocal laser scanning microscopy

**Figure 5:**
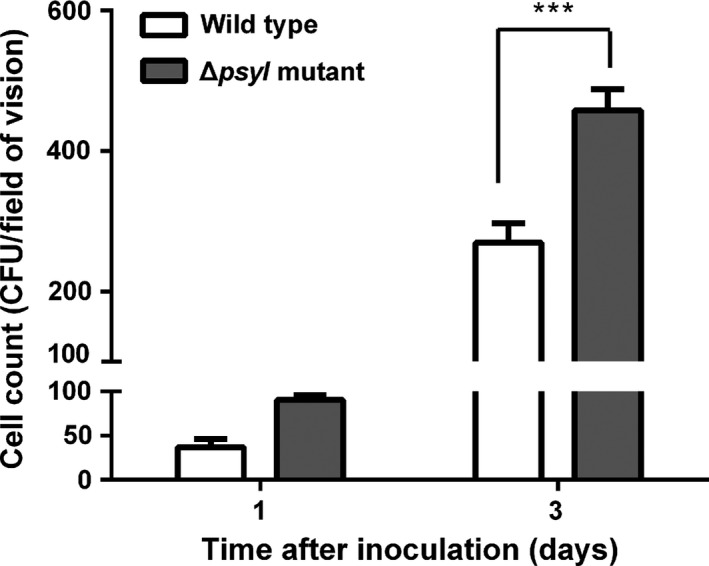
Epiphytic population of *Pseudomonas syringae* 11528 strains. Tobacco leaves were spray‐inoculated with *P. syringae* 11528 wild‐type strain or the Δ*psyI* mutant at the concentrations of 10^6 ^CFU/ml and were observed at 1 and 3 days after inoculation by confocal laser scanning microscopy. Magnification: ×1000 (Bar=20 μm), ×400 (Bar=50 μm)

We also investigated the plant‐colonizing ability of *P. syringae* 11528 using fluorescence‐labeling technology combined with CLSM. GFP‐labeled *P. syringae* 11528 wild‐type and Δ*psyI* mutant strains were constructed and then spray‐inoculated on tobacco leaves with the same inoculum concentration. CLSM observation showed that the majority of both strains assembled in the glandular trichomes at both 1 and 3 days postinoculation (Figure 4). Moreover, we compared the epiphytic populations of viable cells recovered from inoculated tobacco leaves. In contrast to the wild type, the observed population sizes of the Δ*psyI* mutant were larger, especially at 3 days after inoculation (Figure 5). These findings imply that the Δ*psyI* mutant exhibits a larger epiphytic population in inoculated leaves that results and more robust plant colonization ability when compared with the wild type.

#### 3.3.3 Pathogenicity

**Figure 6:**
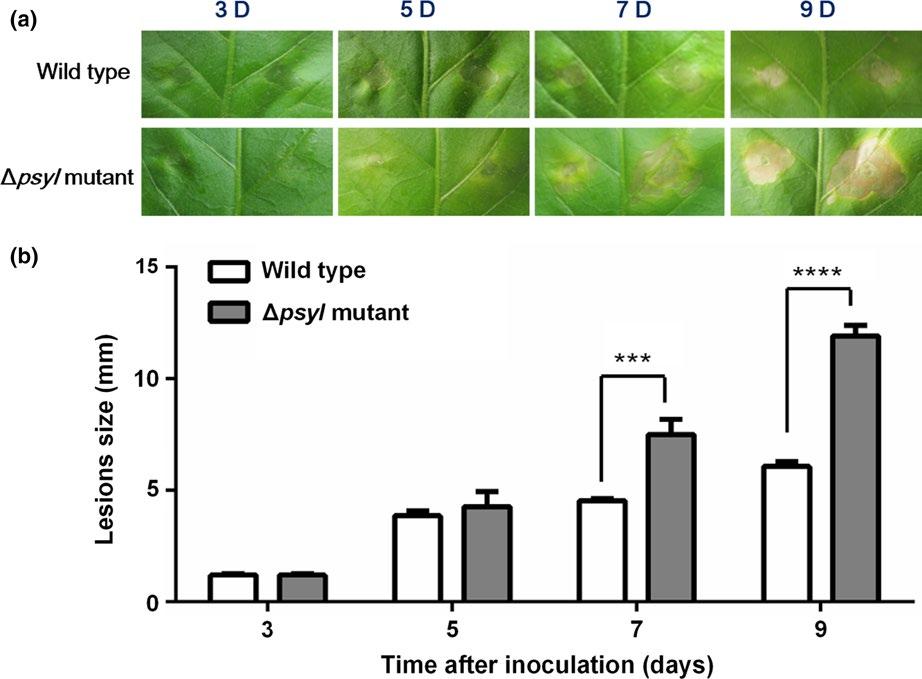
Pathogenicity tests of *Pseudomonas syringae* 11528 strains. Leaf symptoms (a) and lesion sizes (b) on tobacco leaves that were induced by *P. syringae* 11528 wild‐type strain or the Δ*psyI* mutant. Tobacco plants were infiltrated with *P. syringae* 11528 cells at concentrations of 10^8 ^CFU/ml during T phase. Leaf symptoms and lesions sizes were assayed at 3, 5, 7, and 9 days postinoculation under moist conditions at 25°C

To explore the effect of AHL‐dependent regulation in *P. syringae* 11528 on plant infection, pathogenicity tests were conducted via plant inoculation. The wild‐type and Δ*psyI* mutant cells were infiltrated into tobacco leaves and the sizes of necrotic lesion were measured at various time points. Although necrotic leaf tissues were initially observed in tobacco leaves inoculated with either the Δ*psyI* mutant or the wild type at 5 days postinoculation, disease symptoms incited by the Δ*psyI* mutant were more pronounced than that by the wild type (Figure 6a). After 7 days of infection, lesion sizes were significantly larger when tobacco plants were treated with Δ*psyI* mutant than the wild type (Figure 6b). These data indicate that AHL‐mediated QS system affects the interactions between tobacco‐*P. syringae* 11528 and represses disease development.

## 4 DISCUSSION

*P. syringae* 11528 is known to be a phytopathogen that can cause wild‐fire disease in soybeans and tobacco plants. Its QS system possesses *psyI* and *psyR* (Elasri et al., 2001), and the *psyI* is responsible for the AHL biosynthesis. To explore the AHL‐dependent QS regulation on gene expression in *P. syringae* 11528, we compared the transcriptomes of *P. syringae* 11528 Δ*psyI* mutant with those of the wild type at three given time points. A total of 1118 QS‐regulated genes were found to be differentially expressed in the transition from exponential to stationary (T) phase, while dozens of QS‐regulated genes were identified in both L and E phases (Table**** 3). These data demonstrate that the AHL‐mediated QS system may play different roles in transcriptional regulation during *P. syringae* 11528 growth. The clearest effect of QS regulation on gene expression was observed during T phase. Similar to previous studies, the onset of many QS‐dependent processes occurred during the late logarithmic to early stationary phase (Gao et al., 2015; Schuster, Lostroh, Ogi, & Greenberg, 2003). Thus, the regulatory function of the AHL‐mediated QS system is associated with the process of bacterial growth along with the specificity and timing (Schuster et al., 2003). This is consistent with the cell density‐dependent nature of QS, which can regulate the gene expression in response to the growth of bacterial cells (Keller & Surette, 2006). Recently, many studies have carried out transcriptome analysis of plant pathogens to identify QS‐regulated gene expression patterns and have revealed a large number of genes that are subject to QS regulation, such as 6.2% of coding sequences in the *P. aeruginosa* genome (Chugani et al., 2012), 19.6% of coding sequences in *B. glumae* BGR1 genome (Kim et al., 2013), 21.4% of coding sequences in *B. gladioli* BSR3 genome (Kim, Park, Choi, Kim, & Seo, 2014), and 11.5% of coding sequences in *B. glumae* PG1 genome (Gao et al., 2015). In those previous reports, each study focused on an individual organism under different growth conditions, so the amount of genes regulated by QS varied. In *P. syringae* 11528 genome, 6,057 potential protein‐coding genes were identified by Studholme et al. (2009); therefore, we inferred that ~18.5% of those genes were differentially expressed in an AHL‐mediated QS regulatory manner. This finding implies that QS system is a significant regulator on gene expression in *P. syringae* 11528.

For the gene expression levels of those 1118 QS‐regulated genes, the most pronounced regulation was found for genes associated with bacterial flagella and chemotaxis (Table**** 4), which was further confirmed by subsequent GO and pathway enrichment analysis (Figure 1). The bacterial chemotaxis and flagellar assembly pathways were enriched, among which flagellar assembly was completely down‐regulated, and this was consistent with the expression profiles of all genes involved in flagellar assembly in *P. syringae* 11528 wild‐type and Δ*psyI* mutant strains (Figure 2). Bacterial flagella and chemotaxis play important roles in bacterial motility and plant colonization (Alavi et al., 2013; de Weert et al., 2002), processes that allow bacterial cells to seek out better survival environments (Aizawa, 2001). Swarming motility tests confirmed in part the RNA‐seq data, showing that the motility of the Δ*psyI* mutant was significantly enhanced (Figure 3). In the observation of plant colonization, both *P. syringae* 11528 wild‐type and Δ*psyI* mutant cells were mostly viewed in the glandular trichomes (Figure 4). One possible explanation for this observation is that the distribution of cells on leaf surfaces is approximately associated with the spatial heterogeneity of nutrients available on leaf surfaces (Leveau & Lindow, 2001), and glandular trichomes harbor an abundance of nutrients responsible for bacterial survival (Maggi et al., 2010). Similar observations have been obtained in other previous reports (Taiz & Zeiger, 1998). Motility is considered to a virulence factor and is vital for plant–pathogen interactions, because it helps pathogen to locate resources and promotes pathogen invasion into plant tissues (Quiñones et al., 2005). Thus, the ∆*psyI* mutant with more motility possessed a greater ability to access to nutritious sites and a larger population in glandular trichomes at 1 days after inoculation when compared to the wild type (Figure 5). A rapid multiplication of inoculum would occur on tobacco leaves under moist conditions (Quiñones et al., 2005). At 3 days after inoculation, a more significant difference of epiphytic population was observed between the ∆*psyI* mutant and the wild type (Figure 5). A direct correlation between robust motility and a large epiphytic population was obtained in the work of others (Haefele & Lindow, 1987; Lindow, Andersen, & Beattie, 1993), further indicating that motility contributes to epiphytic fitness of phytopathogen on plants. Therefore, the AHL‐dependent regulation in *P. syringae* 11528 does not affect distribution site but repress motility as well as epiphytic growth of this pathogen on plants, resulting in the negative influence of QS system on early plant colonization.

More significant wild‐fire disease symptoms and larger lesion sizes were observed in tobacco leaves treated with *P. syringae* 11528 Δ*psyI* mutant compared with those resulting from treatment with the wild type (Figure 6). There may be two possible explanations for an increase in the severity of disease incited by the Δ*psyI* mutant. During tobacco‐*P. syringae* 11528 interactions, *P. syringae* 11528 acts both as a saprophyte that lives epiphytically on plant surfaces and as a pathogen that resides within the leaf apoplast (Hockett, Burch, & Lindow, 2013). These two states are closely related. The early epiphytic phase is critical for the later course of disease as it provides inoculum for subsequent infection (Hockett et al., 2013; Quiñones et al., 2005). A larger epiphytic population of the Δ*psyI* mutant was observed, presumably resulting in a higher inoculum for later plant infections when compared to the wild type. As an alternative explanation, in addition to genes involved in motility, many genes were negatively regulated in an AHL‐dependent manner, including genes encoding pilus, EPS, secretion systems, and the two‐component system (Table**** 4). Those genes were responsible for bacterial virulence. For example, pilus and EPS contribute to adherence to surfaces and are linked to biofilm formation (Alavi et al., 2013); secretion systems, such as type II, type III, and type VI secretion systems, contribute to bacterial virulence (Bernard, Brunet, Gueguen, & Cascales, 2010; Preston et al., 2005; Studholme et al., 2009), in which the type III secretion system and its TTEs are required for pathogenesis in plants and play critical roles in plant–pathogen interactions (Studholme et al., 2009; Yang, Lee, Cha, & Baik, 2011); and the two‐component system has been reported to regulate the production of EPS as well as motility and is required for virulence in *P. syringae* (Marutani et al., 2008; Willis, Holmstadt, & Kinscherf, 2001). Summarily, the expression of various virulence factors was supppressed by AHL‐dependent QS, resulting in increased virulence of the Δ*psyI* mutant. Thus, due to a larger epiphytic population and being more virulent, the Δ*psyI* mutant caused more serious wild‐fire disease in inoculated tobacco leaves. To initiate pathogenesis, plant pathogenic bacteria must first enter plant tissues and colonize on plants (Melotto et al. 2006). Plant infection was a later stage of plant–pathogen interactions than plant colonization, since wild‐fire disease lesions incited by the Δ*psyI* mutant were obvious only 5 days or more after inoculation. Hence, AHL‐dependent QS regulation in *P. syringae* 11528 may be involved in both early and late stages of disease development. Similar reports revealed that an AhlI‐AhlR QS system in *P. syringae* B728a regulated not only early water soaking but also late tissue maceration in bean plants (Quiñones et al., 2005), but AHL‐mediated QS in *E. carotovora* subsp. atroseptica (*Pectobacterium atrosepticum*) affected late plant infection rather than initial plant colonization (Smadja et al., 2004).

A high sequence identity (85%) is present between the PsyI in *P. syringae* 11528 and LuxI‐homologs (AhlI) in *P. syringae* B728a (Quiñones et al., 2004). Similar AHL‐mediated QS regulation on traits relevant to plant–pathogen interactions exists in these two pathovars, such as negative regulation of swarming motility and the formation of disease lesions, while there is an obvious divergence in regulation of EPS production, a down‐regulation in *P. syringae* 11528 but an up‐regulation in *P. syringae* B728a (Quiñones et al., 2005). Additionally, two virulence traits, iron transport as well as toxin, were postively controlled by QS system in *P. syringae* 11528, while QS regulation is also required for the production of exoenzymes involved in tissue maceration in *P. syringae* B728a (Quiñones et al., 2005). Moreover, Taguchi et al. (2006) have demonstrated that a *psyI* deletion mutant of another *P. syringae* pathovars, *P. syringae* 6605, exhibited enhanced swarming motility and EPS production and decreased siderophore production, biofilm formation, and virulence against tobacco. It seems likely that different microbes possess distinct QS systems and lead to significant variations in the regulation of gene expression, or there may be other regulator(s) which control or coordinate QS system to regulate bacterial activities. In *P. syringae* B728a, AefR and GacA are separate regulators, both of which act as activators of AhlI‐dependent QS system via independent pathways (Quiñones et al., 2004). As proposed by Cha and collaborators, GacA regulon and iron regulon differently affect the AHL production, and both of them coordinately regulate virulence traits related to the pathogenesis of *P. syringae* 11528, and there may be an interaction between them (Cha, Lee, Lee, Oh, & Baik, 2012). Therefore, additional studies are required to further identify other regultors and characterize the interaction between them and PsyI‐dependent QS system in *P. syringae* 11528.

In conclusion, our transcriptional analysis revealed that the most significant regulation of AHL‐mediated QS system on gene expression occurred during T phase in *P. syringae* 11528. A total of 1118 QS‐regulated genes were identified, including numerous genes involved in pathogenicity on plants. Moreover, phenotypic assays revealed that QS‐dependent traits are involved in motility, epiphytic growth, and disease course of plants. These findings suggest that AHL‐regulated traits in *P. syringae* 11528 may be involved in both early plant colonization and late plant infection during plant–pathogen interactions. This study provides insights into the effects of QS‐dependent regulons on the processes of epiphytic growth and virulence of *P. syringae*11528 and extends our understanding of AHL‐mediated QS regulation, including positive and negative regulation, on plant–pathogen interactions.

## CONFLICT OF INTEREST

None declared.

## References

[mbo3440-bib-0001] Aizawa, S.‐I. (2001). Bacterial flagella and type III secretion systems. FEMS Microbiology Letters, 202(2), 157–164. 11520608 10.1111/j.1574-6968.2001.tb10797.x

[mbo3440-bib-0002] Alavi, P. , Muller, H. , Cardinale, M. , Zachow, C. , Sanchez, M. B. , Martinez, J. L. , & Berg, G. (2013). The DSF quorum sensing system controls the positive influence of *Stenotrophomonas maltophilia* on plants. PLoS ONE, 8(7), e67103. 23874407 10.1371/journal.pone.0067103 PMC3715506

[mbo3440-bib-0003] Anders, S. , & Huber, W. (2010). Differential expression analysis for sequence count data. Genome Biology, 11(10), R106. 20979621 10.1186/gb-2010-11-10-r106 PMC3218662

[mbo3440-bib-0005] Baltrus, D. A. , Nishimura, M. T. , Romanchuk, A. , Chang, J. H. , Mukhtar, M. S. , Cherkis, K. , … Dangl, J. L. (2011). Dynamic evolution of pathogenicity revealed by sequencing and comparative genomics of 19 *Pseudomonas syringae* isolates. PLoS Pathogens, 7(7), e1002132. 21799664 10.1371/journal.ppat.1002132 PMC3136466

[mbo3440-bib-0006] Barabote, R. D. , Johnson, O. L. , Zetina, E. , San Francisco, S. K. , Fralick, J. A. , & San Francisco, M. J. (2003). *Erwinia chrysanthemi tolC* is involved in resistance to antimicrobial plant chemicals and is essential for phytopathogenesis. Journal of Bacteriology, 185(19), 5772–5778. 13129948 10.1128/JB.185.19.5772-5778.2003 PMC193971

[mbo3440-bib-0007] Bernard, C. S. , Brunet, Y. R. , Gueguen, E. , & Cascales, E. (2010). Nooks and crannies in type VI secretion regulation. Journal of Bacteriology, 192(15), 3850–3860. 20511495 10.1128/JB.00370-10 PMC2916374

[mbo3440-bib-0008] Brencic, A. , & Winans, S. C. (2005). Detection of and response to signals involved in host‐microbe interactions by plant‐associated bacteria. Microbiology and Molecular Biology Reviews, 69(1), 155–194. 15755957 10.1128/MMBR.69.1.155-194.2005 PMC1082791

[mbo3440-bib-0009] Burse, A. , Weingart, H. , & Ullrich, M. S. (2004). The phytoalexin‐inducible multidrug efflux pump AcrAB contributes virulence in the fire blight pathogen Erwinia amylovora. Molecular Plant‐Microbe Interactions, 17(1), 43–54. 14714867 10.1094/MPMI.2004.17.1.43

[mbo3440-bib-0010] Cha, J. Y. , Lee, D. G. , Lee, J. S. , Oh, J. I. , & Baik, H. S. (2012). GacA directly regulates expression of several virulence genes in *Pseudomonas syringae* pv. *tabaci* 11528. Biochemical and Biophysical Research Communications, 417(2), 665–672. 22166197 10.1016/j.bbrc.2011.11.124

[mbo3440-bib-0011] Cha, J. Y. , Lee, J. S. , Oh, J.‐I. , Choi, J. W. , & Baik, H. S. (2008). Functional analysis of the role of Fur in the virulence of *Pseudomonas syringae* pv. *tabaci* 11528: Fur controls expression of genes involved in quorum‐sensing. Biochemical and Biophysical Research Communications, 366(2), 281–287. 18023417 10.1016/j.bbrc.2007.11.021

[mbo3440-bib-0012] Cheng, F. , Ma, A. , Zhuang, G. , & Fray, R. G. (2016). Exogenous N‐acyl‐homoserine lactones enhance the expression of flagella of pseudomonas syringae and activate defence responses in plants. Molecular Plant Pathology. 10.1111/mpp.12502 PMC6637982 27756102

[mbo3440-bib-0013] Chugani, S. , Kim, B. S. , Phattarasukol, S. , Brittnacher, M. J. , Choi, S. H. , Harwood, C. S. , & Greenberg, E. P. (2012). Strain‐dependent diversity in the *Pseudomonas aeruginosa* quorum‐sensing regulon. Proceedings of the National Academy of Sciences, 109(41), E2823–E2831. 10.1073/pnas.1214128109 PMC3478653 22988113

[mbo3440-bib-0014] Clough, S. J. , Flavier, A. B. , Schell, M. A. , & Denny, T. P. (1997). Differential expression of virulence genes and motility in *Ralstonia* (*Pseudomonas*) *solanacearum* during exponential growth. Applied and Environmental Microbiology, 63(3), 844–850. 16535550 10.1128/aem.63.3.844-850.1997 PMC1389115

[mbo3440-bib-0015] de Weert, S. , Vermeiren, H. , Mulders, I. H. , Kuiper, I. , Hendrickx, N. , Bloemberg, G. V. , … Lugtenberg, B. J. (2002). Flagella‐driven chemotaxis towards exudate components is an important trait for tomato root colonization by *Pseudomonas fluorescens* . Molecular Plant‐Microbe Interactions, 15(11), 1173–1180. 12423023 10.1094/MPMI.2002.15.11.1173

[mbo3440-bib-0016] Deng, X. M. , Zhuang, G. Q. , Ma, A. Z. , Yu, Q. , & Zhuang, X. L. (2014). Construction of a dual fluorescence whole‐cell biosensor to detect N‐acyl homoserine lactones. Journal of Environmental Sciences, 26(2), 415–422. 10.1016/s1001-0742(13)60407-6 25076533

[mbo3440-bib-0018] Dumenyo, C. K. , Mukherjee, A. , Chun, W. , & Chatterjee, A. K. (1998). Genetic and physiological evidence for the production of N‐acyl homoserine lactones by *Pseudomonas syringae* pv. *syringae* and other fluorescent plant pathogenic Pseudomonas species. European Journal of Plant Pathology, 104(6), 569–582.

[mbo3440-bib-0019] Durbin, R. D. (1991). Bacterial phytotoxins: Mechanisms of action. Experientia, 47(8), 776–783.

[mbo3440-bib-0020] Elasri, M. , Delorme, S. , Lemanceau, P. , Stewart, G. , Laue, B. , Glickmann, E. , … Dessaux, Y. (2001). Acyl‐homoserine lactone production is more common among plant‐associated *Pseudomonas* spp. than among soilborne *Pseudomonas* spp. Applied and Environmental Microbiology, 67(3), 1198–1209. 11229911 10.1128/AEM.67.3.1198-1209.2001 PMC92714

[mbo3440-bib-0021] Fuqua, W. C. , Winans, S. C. , & Greenberg, E. P. (1994). Quorum sensing in bacteria: The LuxR‐LuxI family of cell density‐responsive transcriptional regulators. Journal of Bacteriology, 176(2), 269–275. 8288518 10.1128/jb.176.2.269-275.1994 PMC205046

[mbo3440-bib-0022] Gao, R. , Krysciak, D. , Petersen, K. , Utpatel, C. , Knapp, A. , Schmeisser, C. , … Streit, W. R. (2015). Genome‐Wide RNA sequencing analysis of quorum sensing‐controlled regulons in the plant‐associated *Burkholderia glumae* PG1 strain. Applied and Environmental Microbiology, 81(23), 7993–8007. 26362987 10.1128/AEM.01043-15 PMC4651095

[mbo3440-bib-0023] Gasson, M. J. (1980). Indicator technique for antimetabolic toxin production by phytopathogenic species of *Pseudomonas* . Applied and Environmental Microbiology, 39(1), 25–29. 16345492 10.1128/aem.39.1.25-29.1980 PMC291278

[mbo3440-bib-0024] Haefele, D. M. , & Lindow, S. E. (1987). Flagellar motility confers epiphytic fitness advantages upon *Pseudomonas syringae* . Applied and Environmental Microbiology, 53(10), 2528–2533. 16347469 10.1128/aem.53.10.2528-2533.1987 PMC204140

[mbo3440-bib-0025] Heurlier, K. , Denervaud, V. , & Haas, D. (2006). Impact of quorum sensing on fitness of *Pseudomonas aeruginosa* . International Journal of Medical Microbiology, 296(2–3), 93–102. 16503417 10.1016/j.ijmm.2006.01.043

[mbo3440-bib-0026] Hockett, K. L. , Burch, A. Y. , & Lindow, S. E. (2013). Thermo‐regulation of genes mediating motility and plant interactions in *Pseudomonas syringae* . PLoS ONE, 8(3), e59850. 23527276 10.1371/journal.pone.0059850 PMC3602303

[mbo3440-bib-0027] Jakob, K. , Kniskern, J. M. , & Bergelson, J. (2007). The role of pectate lyase and the jasmonic acid defense response in *Pseudomonas viridiflava* virulence. Molecular Plant‐Microbe Interactions, 20(2), 146–158. 17313166 10.1094/MPMI-20-2-0146

[mbo3440-bib-0028] Jones, S. , Yu, B. , Bainton, N. A. , Birdsall, M. , Bycroft, B. , Chhabra, S. , … Stephens, S. (1993). The lux autoinducer regulates the production of exoenzyme virulence determinants in *Erwinia carotovora* and *Pseudomonas aeruginosa* . The EMBO Journal, 12(6), 2477–2482. 8508773 10.1002/j.1460-2075.1993.tb05902.x PMC413484

[mbo3440-bib-0030] Keller, L. , & Surette, M. G. (2006). Communication in bacteria: An ecological and evolutionary perspective. Nature Reviews Microbiology, 4(4), 249–258. 16501584 10.1038/nrmicro1383

[mbo3440-bib-0031] Kim, S. , Park, J. , Choi, O. , Kim, J. , & Seo, Y. S. (2014). Investigation of quorum sensing‐dependent gene expression in *Burkholderia gladioli* BSR3 through RNA‐seq analyses. Journal of Microbiology Biotechnology, 24(12), 1609–1621. 25223327 10.4014/jmb.1408.08064

[mbo3440-bib-0032] Kim, S. , Park, J. , Kim, J. H. , Lee, J. , Bang, B. , Hwang, I. , & Seo, Y. S. (2013). RNAseq‐based transcriptome analysis of *Burkholderia glumae* quorum sensing. The Plant Pathology Journal, 29(3), 249–259. 25288952 10.5423/PPJ.OA.04.2013.0044 PMC4174805

[mbo3440-bib-0033] Langmead, B. , & Salzberg, S. L. (2012). Fast gapped‐read alignment with Bowtie 2. Nature Methods, 9(4), 357–359. 22388286 10.1038/nmeth.1923 PMC3322381

[mbo3440-bib-0034] Leveau, J. H. , & Lindow, S. E. (2001). Appetite of an epiphyte: Quantitative monitoring of bacterial sugar consumption in the phyllosphere. Proceedings of the National Academy of Sciences, 98(6), 3446–3453. 10.1073/pnas.061629598 PMC30673 11248098

[mbo3440-bib-0035] Lindeberg, M. , Myers, C. R. , Collmer, A. , & Schneider, D. J. (2008). Roadmap to new virulence determinants in *Pseudomonas syringae*: Insights from comparative genomics and genome organization. Molecular Plant‐Microbe Interactions, 21(6), 685–700. 18624633 10.1094/MPMI-21-6-0685

[mbo3440-bib-0036] Lindow, S. E. , Andersen, G. , & Beattie, G. A. (1993). Characteristics of insertional mutants of *Pseudomonas syringae* with reduced epiphytic fitness. Applied and Environmental Microbiology, 59(5), 1593–1601. 16348939 10.1128/aem.59.5.1593-1601.1993 PMC182124

[mbo3440-bib-0037] Loh, J. , Pierson, E. A. , Pierson, L. S. , Stacey, G. , & Chatterjee, A. (2002). Quorum sensing in plant‐associated bacteria. Current Opinion in Plant Biology, 5(4), 285–290. 12179960 10.1016/s1369-5266(02)00274-1

[mbo3440-bib-0038] Lv, D. , Ma, A. Z. , Tang, X. M. , Bai, Z. H. , Qi, H. Y. , & Zhuang, G. Q. (2013). Profile of the culturable microbiome capable of producing acyl‐homoserine lactone in the tobacco phyllosphere. Journal of Environmental Sciences, 25(2), 357–366. 10.1016/s1001-0742(12)60027-8 23596957

[mbo3440-bib-0039] Mäe, A. , Montesano, M. , Koiv, V. , & Palva, E. T. (2001). Transgenic plants producing the bacterial pheromone *N*‐acyl‐homoserine lactone exhibit enhanced resistance to the bacterial phytopathogen *Erwinia carotovora* . Molecular Plant‐Microbe Interactions, 14(9), 1035–1042. 11551068 10.1094/MPMI.2001.14.9.1035

[mbo3440-bib-0040] Maggi, F. , Papa, F. , Cristalli, G. , Sagratini, G. , Vittori, S. , & Giuliani, C. (2010). Histochemical localization of secretion and composition of the essential oil in *Melittis melissophyllum* L. subsp. *melissophyllum* from Central Italy. Flavour and Fragrance Journal, 25(2), 63–70.

[mbo3440-bib-0041] Mao, X. , Tao, C. J. G. O. , & Wei, L. (2005). Automated genome annotation and pathway identification using the KEGG Orthology (KO) as a controlled vocabulary. Bioinformatics, 21(19), 3787–3793. 15817693 10.1093/bioinformatics/bti430

[mbo3440-bib-0042] Marutani, M. , Taguchi, F. , Ogawa, Y. , Hossain, M. M. , Inagaki, Y. , Toyoda, K. , … Ichinose, Y. (2008). Gac two‐component system in *Pseudomonas syringae* pv. *tabaci* is required for virulence but not for hypersensitive reaction. Molecular Genetics and Genomics, 279(4), 313–322. 18080141 10.1007/s00438-007-0309-y

[mbo3440-bib-0043] McLean, R. J. , Whiteley, M. , Stickler, D. J. , & Fuqua, W. C. (1997). Evidence of autoinducer activity in naturally occurring biofilms. FEMS Microbiology Letters, 154(2), 259–263. 9311122 10.1111/j.1574-6968.1997.tb12653.x

[mbo3440-bib-1000] Melotto, M. , Underwood, W. , Koczan, J. , Nomura, K. , Sheng, Y. H. (2006) Plant Stomata Function in Innate Immunity against Bacterial Invasion. Cell 126(5):969‐980. 16959575 10.1016/j.cell.2006.06.054

[mbo3440-bib-0045] Mortazavi, A. , Williams, B. A. , McCue, K. , Schaeffer, L. , & Wold, B. (2008). Mapping and quantifying mammalian transcriptomes by RNA‐Seq. Nature Methods, 5(7), 621–628. 18516045 10.1038/nmeth.1226 PMC13303166

[mbo3440-bib-0048] Pirhonen, M. , Flego, D. , Heikinheimo, R. , & Palva, E. T. (1993). A small diffusible signal molecule is responsible for the global control of virulence and exoenzyme production in the plant pathogen *Erwinia carotovora* . The EMBO Journal, 12(6), 2467–2476. 8508772 10.1002/j.1460-2075.1993.tb05901.x PMC413482

[mbo3440-bib-0049] Preston, G. M. , Studholme, D. J. , & Caldelari, I. (2005). Profiling the secretomes of plant pathogenic Proteobacteria. FEMS Microbiology Reviews, 29(2), 331–360. 15808747 10.1016/j.femsre.2004.12.004

[mbo3440-bib-0050] Quiñones, B. , Dulla, G. , & Lindow, S. E. (2005). Quorum sensing regulates exopolysaccharide production, motility, and virulence in *Pseudomonas syringae* . Molecular Plant‐Microbe Interactions, 18(7), 682–693. 16042014 10.1094/MPMI-18-0682

[mbo3440-bib-0051] Quiñones, B. , Pujol, C. J. , & Lindow, S. E. (2004). Regulation of AHL production and its contribution to epiphytic fitness in *Pseudomonas syringae* . Molecular Plant‐Microbe Interactions, 17(5), 521–531. 15141956 10.1094/MPMI.2004.17.5.521

[mbo3440-bib-0052] Rahme, L. G. , Ausubel, F. M. , Cao, H. , Drenkard, E. , Goumnerov, B. C. , Lau, G. W. , … Tompkins, R. G. (2000). Plants and animals share functionally common bacterial virulence factors. Proceedings of the National Academy of Sciences, 97(16), 8815–8821. 10.1073/pnas.97.16.8815 PMC34017 10922040

[mbo3440-bib-0053] Ribeiro, R. , Hagedorn, D. , Durbin, R. , & Uchytil, T. (1979). Characterization of the bacterium inciting bean wildfire in Brazil. Phytopathology, 69(3), 208–212.

[mbo3440-bib-1001] Schuster, M. , Greenberg, E. P . (2006) A network of networks: quorum‐sensing gene regulation in Pseudomonas aeruginosa. International Journal of Medical Microbiology 296(2‐3):73‐81. 16476569 10.1016/j.ijmm.2006.01.036

[mbo3440-bib-0054] Schuster, M. , Lostroh, C. P. , Ogi, T. , & Greenberg, E. P. (2003). Identification, timing, and signal specificity of *Pseudomonas aeruginosa* quorum‐controlled genes: A transcriptome analysis. Journal of Bacteriology, 185(7), 2066–2079. 12644476 10.1128/JB.185.7.2066-2079.2003 PMC151497

[mbo3440-bib-0055] Shaw, P. D. , Ping, G. , Daly, S. L. , Cha, C. , Cronan, J. E. , Rinehart, K. L. , & Farrand, S. K. (1997). Detecting and characterizing *N*‐acyl‐homoserine lactone signal molecules by thin‐layer chromatography. Proceedings of the National Academy of Sciences of the United States of America, 94(12), 6036–6041. 9177164 10.1073/pnas.94.12.6036 PMC20996

[mbo3440-bib-0056] Smadja, B. , Latour, X. , Faure, D. , Chevalier, S. , Dessaux, Y. , & Orange, N. (2004). Involvement of *N*‐acylhomoserine lactones throughout plant infection by *Erwinia carotovora* subsp. *atroseptica* (*Pectobacterium atrosepticum*). Molecular Plant‐Microbe Interactions, 17(11),1269–1278. 15553252 10.1094/MPMI.2004.17.11.1269

[mbo3440-bib-0057] Smith, R. (2003). *P. aeruginosa* quorum‐sensing systems and virulence. Current Opinion in Microbiology, 6(1), 56–60. 12615220 10.1016/s1369-5274(03)00008-0

[mbo3440-bib-0058] Steindler, L. , & Venturi, V. (2007). Detection of quorum‐sensing *N*‐acyl homoserine lactone signal molecules by bacterial biosensors. FEMS Microbiology Letter, 266(1), 1–9. 10.1111/j.1574-6968.2006.00501.x 17233715

[mbo3440-bib-0059] Stiner, L. , & Halverson, L. J. (2002). Development and characterization of a green fluorescent protein‐based bacterial biosensor for bioavailable toluene and related compounds. Applied and Environmental Microbiology, 68(4), 1962–1971. 11916719 10.1128/AEM.68.4.1962-1971.2002 PMC123894

[mbo3440-bib-0060] Studholme, D. J. , Ibanez, S. G. , MacLean, D. , Dangl, J. L. , Chang, J. H. , & Rathjen, J. P. (2009). A draft genome sequence and functional screen reveals the repertoire of type III secreted proteins of *Pseudomonas syringae* pathovar *tabaci* 11528. BMC Genomics, 10(1), 395. 19703286 10.1186/1471-2164-10-395 PMC2745422

[mbo3440-bib-0062] Taguchi, F. , Ogawa, Y. , Takeuchi, K. , Suzuki, T. , Toyoda, K. , Shiraishi, T. , & Ichinose, Y. (2006). A homologue of the 3‐oxoacyl‐(acyl carrier protein) synthase III gene located in the glycosylation Island of *Pseudomonas syringae* pv. *tabaci* regulates virulence factors via *N*‐acyl homoserine lactone and fatty acid synthesis. Journal of Bacteriology, 188(24), 8376–8384. 17028280 10.1128/JB.00763-06 PMC1698239

[mbo3440-bib-0063] Taguchi, F. , Shibata, S. , Suzuki, T. , Ogawa, Y. , Aizawa, S.‐I. , Takeuchi, K. , & Ichinose, Y. (2008). Effects of glycosylation on swimming ability and flagellar polymorphic transformation in *Pseudomonas syringae* pv. *tabaci* 6605. Journal of Bacteriology, 190(2), 764–768. 18024523 10.1128/JB.01282-07 PMC2223687

[mbo3440-bib-0064] Taiz, L. , & Zeiger, E. (1998). Mineral nutrition. Plant Physiology, 2, 103–124.

[mbo3440-bib-0065] Thomas, M. D. , Langstonunkefer, P. J. , Uchytil, T. F. , & Durbin, R. D. (1983). Inhibition of glutamine‐synthetase from pea by tabtoxinine‐beta‐lactam. Plant Physiology, 71(4), 912–915. 16662928 10.1104/pp.71.4.912 PMC1066143

[mbo3440-bib-0066] Von Bodman, S. B. , Bauer, W. D. , & Coplin, D. L. (2003). Quorum sensing in plant‐pathogenic bacteria. Annual Review of Phytopathology, 41(1), 455–482. 10.1146/annurev.phyto.41.052002.095652 12730390

[mbo3440-bib-0067] Von Bodman, S. B. , & Farrand, S. K. (1995). Capsular polysaccharide biosynthesis and pathogenicity in *Erwinia stewartii* require induction by an *N*‐acylhomoserine lactone autoinducer. Journal of Bacteriology, 177(17), 5000–5008. 7665477 10.1128/jb.177.17.5000-5008.1995 PMC177277

[mbo3440-bib-0068] Von Bodman, S. B. , Majerczak, D. R. , & Coplin, D. L. (1998). A negative regulator mediates quorum‐sensing control of exopolysaccharide production in *Pantoea stewartii* subsp. *stewartii* . Proceedings of the National Academy of Sciences, 95(13), 7687–7692. 10.1073/pnas.95.13.7687 PMC22724 9636211

[mbo3440-bib-0069] Waters, C. M. , & Bassler, B. L. (2005). Quorum sensing: Cell‐to‐cell communication in bacteria. Annual Review of Cell and Developmental Biology, 21, 319–346. 10.1146/annurev.cellbio.21.012704.131001 16212498

[mbo3440-bib-0070] Whitehead, N. A. , Barnard, A. M. L. , Slater, H. , Simpson, N. J. L. , & Salmond, G. P. C. (2001). Quorum‐sensing in Gram‐negative bacteria. FEMS Microbiology Reviews, 25(4), 365–404. 11524130 10.1111/j.1574-6976.2001.tb00583.x

[mbo3440-bib-0071] Willis, D. K. , Holmstadt, J. J. , & Kinscherf, T. G. (2001). Genetic evidence that loss of virulence associated with *gacS* or *gacA* mutations in *Pseudomonas syringae* B728a does not result from effects on alginate production. Applied and Environmental Microbiology, 67(3), 1400–1403. 11229941 10.1128/AEM.67.3.1400-1403.2001 PMC92744

[mbo3440-bib-0072] Yang, H. J. , Lee, J. S. , Cha, J. Y. , & Baik, H. S. (2011). Negative regulation of pathogenesis in *Pseudomonas syringae* pv. *tabaci* 11528 by ATP‐dependent lon protease. Molecules and cells, 32(4), 317–323. 21904881 10.1007/s10059-011-1017-3 PMC3887642

[mbo3440-bib-0073] Yao, J. , & Allen, C. (2006). Chemotaxis is required for virulence and competitive fitness of the bacterial wilt pathogen *Ralstonia solanacearum* . Journal of Bacteriology, 188(10), 3697–3708. 16672623 10.1128/JB.188.10.3697-3708.2006 PMC1482862

[mbo3440-bib-0074] Young, M. D. , Wakefield, M. J. , Smyth, G. K. , & Oshlack, A. (2010). Method Gene ontology analysis for RNA‐seq: Accounting for selection bias. Genome Biology, 11, R14. 20132535 10.1186/gb-2010-11-2-r14 PMC2872874

