# Supplementary material for: N‐acylhomoserine lactone‐regulation of genes mediating motility and pathogenicity in Pseudomonas syringae pathovar tabaci 11528
Source: Microbiologyopen. 2017 Jan 29;6(3):e00440. doi: 10.1002/mbo3.440 (PMC5458577; doi:10.1002/mbo3.440)
Supplement: Supplementary file 1 [file MBO3-6-na-s001.pdf]

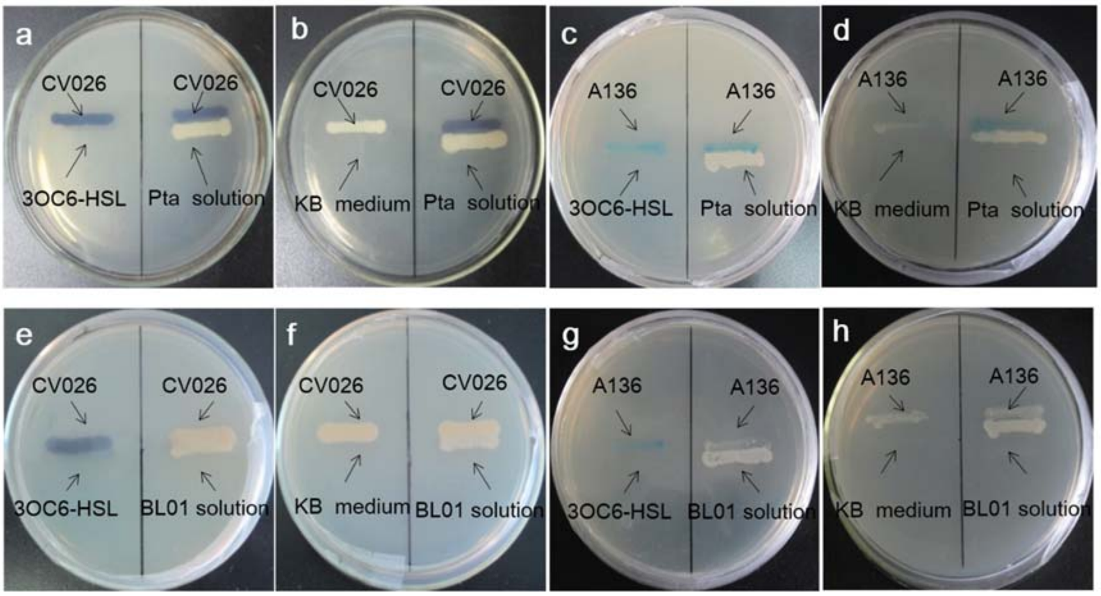

Fig. S1 Representative bioassays for AHL production in *P. syringae* 11528 strains. Solution of *P. syringae* wild-type strain (Pta) (a-d) and △*psyI* mutant (BL01) (e-h) were tested using biosensor strains *C. violaceum* CV026 and *A. tumefaciens* A136, respectively. *N*-(3-oxo-hexanoyl)-L-HSL (3OC6-HSL) (100 nM) and King's medium B (KB) medium were used as positive and negative controls, respectively.

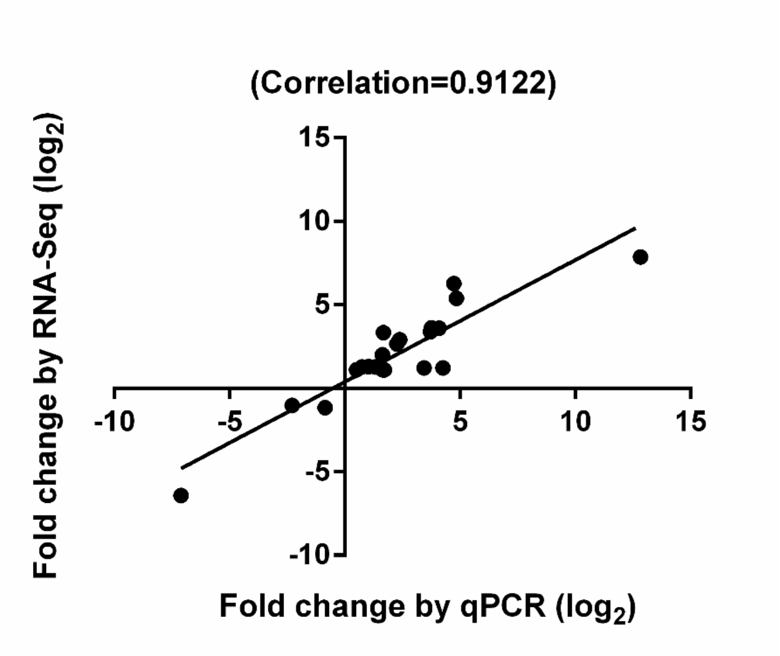

Fig. S2 Validation of RNA-seq results using qPCR. Each fold change of the RNA-seq data (log_2) for 10 randomly selected genes was compared with the fold change from qPCR (log_2).

Table S1 Primers for validating RNA-seq data in this study

Primers/ Gene locus	Forward primer (5'–3')	Reverse primer(5'–3')
PSYTB_00949	ATGGCGTTTGCAGAGCTACT	CCCGTGTTCGATTCTGTCCA
PSYTB_00954	GTATATTGAGCGCCGAGGAGC	CAGCCCAAAAGCCTGAGCGC
PSYTB_00959	CTGCGGCTGATTCGTGTGACTAA	CGGAGAAAGGTCGAGCGATT
PSYTB_07791	GGCATGATCAACGCACTGGAA	GTACGGCCGGAGTGAATGAC
PSYTB_11508	CACTTTCGGTGACGCAACTTGC	CTCGGCCAGCCACTTGCG
PSYTB_13500	GAACCGCATACGCTGATCCG	GCTGACAATGTGATGGGTAAACG
PSYTB_17275	GGACTACGTGCCGACCAACAAAC	CATGAAATCCTGTACCGCACCG
PSYTB_18604	GCGATCTGTCTGGTGCTGG	GACTGCAACAACACGATCCAGG
PSYTB_18609	GACCTGGTAGAAGTCGAATCGCC	GTCGGCGTTGTAGGCCAGCA
PSYTB_18614	GCGATATCGGCATGGTGTTCC'	CACGCGCTGCTGCTGGCCA
PSYTB_18991	GGTAGGCAGCATCGTGATTGTCG	GATGGAACGGCGACAGGCTCAT
PSYTB_18996	GGACCTTGAAACCAGCGGCTTGA	GCAATCGCACTGGGCGCCAAA
PSYTB_23381	GCACTCGTCGGCAGCATTTTCACC	GCTCGGCGGCGGTATTGATTTG
PSYTB_23386	GGCTCAGAACATGGCAGATGAGGG	GATACAGCGGCTCGGCTTCGTTG
PSYTB_23396	GATTCTGATCGGCTACGGACTGG	GGTATGGTCGAGCAGTTGCGGC
PSYTB_23406	GGACGTGACCTTTGCCAGTGTG	GCACCAGCTGGTTATCCATACCA
PSYTB_23411	GCTTTATCGAAGTCAGCCGCACC	CGCCGCCCCAGACAATATTGAAAT
PSYTB_23581	GGAGGTACTGGACTTTGCCCTG	GAAGATATCCAGATGCGTCGCG
PSYTB_23591	GTTCTTCAAGGCGGCCGGT	GAGGGTGGCGCATTTGGTGTCT
PSYTB_23596	GAACAGTCTATATGCCGGTATC	GCAGGCTGGCGACGATCATG
PSYTB_23601	CAACACCTGTGATCCCTACATGC	CATTTCGGTCAGTGGTGCGTGC
PSYTB_23616	GGCTCAGAACATGGCAGATGAGGG	GATACAGCGGCTCGGCTTCGTTG
16S rRNA	GCTCGTGTCGTGAGATGTT	TGTAGCCCAGGTCATAAGG

Table S2 The complete list of QS-regulated genes in *P. syringae* pv. *tabaci* 11528. The minus sign before fold change corresponds to down-regulation of the corresponding genes in the wild-type strain.

Gene locus	Predicted function	Fold change (Wild type/Δ*psyI* mutant)
PSYTB_23591	hypothetical protein	348.28
PSYTB_23596	—	268.45
PSYTB_23586	hypothetical protein	241.72
PSYTB_23601	acyl-homoserine-lactone synthase	204.49
PSYTB_22135	acetyl-coenzyme A synthetase	136.00
PSYTB_23581	dihydrodipicolinate synthase family protein	131.79
PSYTB_18991	cyclic nucleotide-binding protein	119.38
PSYTB_23576	hypothetical protein	106.62
PSYTB_23571	—	83.92
PSYTB_11558	cation acetate symporter	74.64
PSYTB_23621	pyruvate dehydrogenase	67.30
PSYTB_11553	membrane protein	63.41
PSYTB_18614	spermidine/putrescine ABC transporterATP-binding protein	39.18
PSYTB_18996	DNA polymerase III subunit epsilon	38.69
PSYTB_23611	pyruvate dehydrogenase	33.89
PSYTB_18609	spermidine/putrescine ABC transportersubstrate-binding protein	30.72
PSYTB_18604	polyamine ABC transporter substrate-bindingprotein	28.27
PSYTB_09031	aldehyde dehydrogenase	25.03
PSYTB_20676	urocanate hydratase	24.45
PSYTB_18599	polyamine ABC transporter permease	21.05
PSYTB_10108	membrane protein	18.18
PSYTB_03826	porin	17.67
PSYTB_23606	LuxR family transcriptional regulator	15.95
PSYTB_17275	carbon starvation protein A	15.37
PSYTB_17280	hypothetical protein	14.94
PSYTB_17989	fagA protein	14.59
PSYTB_20681	histidine ABC transporter substrate-bindingprotein	13.25
PSYTB_10113	C4-dicarboxylate ABC transportersubstrate-binding protein	12.55
PSYTB_11508	hypothetical protein	12.13
PSYTB_20436	integration host factor	12.04
PSYTB_12433	GntR family transcriptional regulator	11.66
PSYTB_10103	tripartite tricarboxylate transporter TctA	11.54
PSYTB_20551	porin	10.84

Gene locus	Predicted function	Fold change (Wild type/Δ*psyI* mutant)
PSYTB_04715	amino acid ABC transporter substrate-bindingprotein	10.75
PSYTB_03806	C4-dicarboxylate ABC transporter	10.46
PSYTB_19626	hypothetical protein	10.43
PSYTB_19016	AraC family transcriptional regulator	9.72
PSYTB_17545	hypothetical protein	9.66
PSYTB_12423	hypothetical protein	9.53
PSYTB_12610	LacI family transcriptional regulator	9.33
PSYTB_04720	amino acid ABC transporter permease	9.08
PSYTB_25229	hypothetical protein	9.05
PSYTB_16570	rhizopine-binding protein	8.72
PSYTB_00200	—	8.59
PSYTB_20686	ABC transporter permease	8.29
PSYTB_06736	citrate transporter	8.08
PSYTB_15800	hypothetical protein	7.98
PSYTB_04725	amino acid ABC transporter permease	7.91
PSYTB_20696	histidine ammonia-lyase	7.90
PSYTB_12605	sugar ABC transporter ATP-binding protein	7.73
PSYTB_07306	hypothetical protein	7.70
PSYTB_17500	5-dehydro-4-deoxyglucarate dehydratase	7.40
PSYTB_19681	2-succinyl-6-hydroxy-2,4-cyclohexadiene-1-carboxylate synthase	7.36
PSYTB_20691	ABC transporter ATP-binding protein	7.33
PSYTB_12428	3-ketoacyl-ACP reductase	7.27
PSYTB_15270	MFS transporter	7.10
PSYTB_07311	bacteriocin immunity protein	6.94
PSYTB_07291	sn-glycerol-3-phosphate transporter	6.92
PSYTB_26296	sensor histidine kinase	6.84
PSYTB_18574	esterase	6.76
PSYTB_19126	amino acid ABC transporter substrate-bindingprotein	6.72
PSYTB_10423	ABC transporter	6.71
PSYTB_06741	—	6.60
PSYTB_04730	arginine ABC transporter ATP-binding protein	6.33
PSYTB_25054	hypothetical protein	6.27
PSYTB_03816	NAD-dependent dehydratase	6.06
PSYTB_12600	ABC transporter permease	6.05
PSYTB_09436	transcriptional regulator	6.05
PSYTB_04170	malate:quinone oxidoreductase	5.88
PSYTB_07261	GntR family transcriptional regulator	5.85
PSYTB_16595	hypothetical protein	5.76
PSYTB_29095	—	5.72
PSYTB_07316	bacteriocin immunity protein	5.65
PSYTB_03801	C4-dicarboxylate ABC transporter	5.61

Gene locus	Predicted function	Fold change (Wild type/Δ*psyI* mutant)
PSYTB_08306	membrane protein	5.61
PSYTB_12418	calcium-binding protein	5.35
PSYTB_16530	myo-inosose-2 dehydratase	5.33
PSYTB_27732	hypothetical protein	5.31
PSYTB_18149	AraC family transcriptional regulator	5.29
PSYTB_08931	glycosyl transferase family 2	5.29
PSYTB_02194	ABC transporter substrate-binding protein	5.28
PSYTB_20701	histidine ammonia-lyase	5.24
PSYTB_17994	class II fumarate hydratase	5.23
PSYTB_10118	hypothetical protein	5.16
PSYTB_00959	transcriptional regulator	5.13
PSYTB_02184	ABC transporter permease	5.10
PSYTB_02189	amino acid ABC transporter permease	4.91
PSYTB_03811	gluconolactonase	4.87
PSYTB_03239	type VI secretion protein	4.84
PSYTB_13465	galactonate dehydratase	4.79
PSYTB_13460	D-galactonate transporter	4.79
PSYTB_03821	—	4.73
PSYTB_08311	hypothetical protein	4.66
PSYTB_09796	—	4.64
PSYTB_16525	5-dehydro-2-deoxygluconokinase	4.55
PSYTB_13806	isocitrate dehydrogenase	4.50
PSYTB_01769	enolase	4.43
PSYTB_02364	isovaleryl-CoA dehydrogenase	4.40
PSYTB_10593	ABC transporter substrate-binding protein	4.38
PSYTB_14840	AsnC family transcriptional regulator	4.38
PSYTB_03796	membrane protein	4.36
PSYTB_02534	sugar ABC transporter permease	4.32
PSYTB_13470	2-dehydro-3-deoxy-6-phosphogalactonate aldolase	4.32
PSYTB_06726	cupin	4.29
PSYTB_01784	sugar ABC transporter substrate-binding protein	4.29
PSYTB_06716	—	4.26
PSYTB_02239	6-phosphogluconolactonase	4.24
PSYTB_03299	LysR family transcriptional regulator	4.23
PSYTB_27722	AsnC family transcriptional regulator	4.19
PSYTB_17540	membrane protein	4.19
PSYTB_02369	methylcrotonoyl-CoA carboxylase	4.16
PSYTB_05005	sugar ABC transporter permease	4.16
PSYTB_02529	sugar ABC transporter substrate-binding protein	4.08
PSYTB_21175	HxlR family transcriptional regulator	4.05
PSYTB_12595	LacI family transcriptional regulator	4.04
PSYTB_01689	hypothetical protein	3.97

Gene locus	Predicted function	Fold change (Wild type/Δ*psyI* mutant)
PSYTB_09216	iron dicitrate transporter	3.96
PSYTB_06572	—	3.95
PSYTB_05020	porin	3.93
PSYTB_07401	hexuronate transporter ExuT	3.87
PSYTB_16190	permease	3.87
PSYTB_09546	—	3.86
PSYTB_16545	TIM alpha/beta barrel protein	3.84
PSYTB_14093	GntR family transcriptional regulator	3.83
PSYTB_09226	iron siderophore-binding protein	3.82
PSYTB_06731	hypothetical protein	3.82
PSYTB_19336	hybrid-cluster NAD(P)-dependent oxidoreductase	3.77
PSYTB_05385	MFS transporter	3.75
PSYTB_30537	—	3.75
PSYTB_26635	soluble pyridine nucleotide transhydrogenase	3.72
PSYTB_01694	hypothetical protein	3.72
PSYTB_08316	hypothetical protein	3.70
PSYTB_13500	LysR family transcriptional regulator	3.66
PSYTB_00180	dehydrogenase	3.65
PSYTB_22583	50S ribosomal protein L7/L12	3.64
PSYTB_07326	colicin	3.61
PSYTB_22578	50S ribosomal protein L10	3.60
PSYTB_25289	DNA invertase	3.59
PSYTB_19471	cytochrome C	3.56
PSYTB_20996	mRNA interferase	3.54
PSYTB_18739	diguanylate phosphodiesterase	3.54
PSYTB_28427	hypothetical protein	3.53
PSYTB_00739	nucleoside hydrolase	3.52
PSYTB_20521	LysR family transcriptional regulator	3.50
PSYTB_02544	sugar ABC transporter ATP-binding protein	3.49
PSYTB_02539	mannitol ABC transporter permease	3.47
PSYTB_18976	malate synthase G	3.46
PSYTB_25601	hypothetical protein	3.46
PSYTB_00275	D-xylose ABC transporter, ATP-binding protein	3.46
PSYTB_14353	mandelate racemase	3.44
PSYTB_12590	ribokinase	3.44
PSYTB_04175	—	3.43
PSYTB_14333	peptide ABC transporter ATP-binding protein	3.43
PSYTB_27737	plasmid stabilization protein	3.41
PSYTB_27312	sugar transporter	3.40
PSYTB_17455	porin	3.39
PSYTB_20561	hypothetical protein	3.38
PSYTB_25494	outer membrane protein	3.37

Gene locus	Predicted function	Fold change (Wild type/Δ*psyI* mutant)
PSYTB_00195	fatty acid desaturase	3.37
PSYTB_23951	ABC transporter ATP-binding protein	3.36
PSYTB_02179	arginine ABC transporter ATP-binding protein	3.35
PSYTB_10728	branched chain amino acid ABC transportersubstrate-binding protein	3.34
PSYTB_04825	cold-shock protein	3.32
PSYTB_12118	gluconolactonase	3.32
PSYTB_00350	acyl-CoA synthetase	3.29
PSYTB_25024	ABC transporter substrate-binding protein	3.29
PSYTB_08861	branched-chain amino acid transporter permeasesubunit LivH	3.28
PSYTB_06117	adenylate kinase	3.28
PSYTB_03004	arabinose ABC transporter substrate-bindingprotein	3.27
PSYTB_19436	lysine transporter LysE	3.26
PSYTB_17465	ABC transporter	3.24
PSYTB_14173	MFS transporter	3.24
PSYTB_16240	4-hydroxyphenylpyruvate dioxygenase	3.24
PSYTB_19601	hypothetical protein	3.21
PSYTB_28477	hypothetical protein	3.21
PSYTB_02969	transcriptional regulator	3.21
PSYTB_28002	amidinotransferase	3.19
PSYTB_09801	hypothetical protein	3.17
PSYTB_13475	2-dehydro-3-deoxygalactonokinase	3.16
PSYTB_23976	ABC transporter substrate-binding protein	3.16
PSYTB_22796	integrase	3.15
PSYTB_19371	hypothetical protein	3.14
PSYTB_10583	histidine/lysine/arginine/ornithine ABCtransporter permease HisM	3.14
PSYTB_24262	—	3.12
PSYTB_12168	nickel ABC transporter substrate-bindingprotein	3.12
PSYTB_20236	cell wall assembly protein	3.11
PSYTB_12388	sugar ABC transporter	3.11
PSYTB_16535	5-deoxy-glucuronate isomerase	3.11
PSYTB_12715	membrane protein	3.09
PSYTB_06941	AsnC family transcriptional regulator	3.07
PSYTB_11283	hypothetical protein	3.07
PSYTB_13390	type II citrate synthase	3.06
PSYTB_20886	RHS repeat-associated core domain-containingprotein	3.06
PSYTB_03998	50S ribosomal protein L25	3.03
PSYTB_20431	pyridine nucleotide-disulfide oxidoreductase	3.02
PSYTB_16055	class C beta-lactamase	3.01
PSYTB_16555	inositol 2-dehydrogenase	3.01

Gene locus	Predicted function	Fold change (Wild type/Δ*psyI* mutant)
PSYTB_04775	dipicolinate synthase	2.99
PSYTB_23971	—	2.99
PSYTB_24312	muramidase	2.98
PSYTB_09751	binary cytotoxin component	2.97
PSYTB_02374	gamma-carboxygeranoyl-CoA hydratase	2.97
PSYTB_14795	acetyl-CoA acetyltransferase	2.97
PSYTB_00310	hypothetical protein	2.96
PSYTB_12750	protocatechuate 3,4-dioxygenase subunit beta	2.95
PSYTB_24512	phosphoenolpyruvate carboxykinase	2.93
PSYTB_02379	3-methylcrotonyl-CoA carboxylase subunit alpha	2.93
PSYTB_21835	RND transporter	2.93
PSYTB_08076	hemolysin D	2.93
PSYTB_02999	L-arabinose transporter ATP-binding protein	2.92
PSYTB_20516	NAD(P) transhydrogenase subunit alpha	2.91
PSYTB_20766	—	2.91
PSYTB_17415	hypothetical protein	2.91
PSYTB_08856	branched chain amino acid ABC transportersubstrate-binding protein	2.90
PSYTB_14098	MFS transporter	2.90
PSYTB_15780	hypothetical protein	2.90
PSYTB_00280	xylose ABC transporter substrate-bindingprotein	2.89
PSYTB_10098	membrane protein	2.88
PSYTB_07851	glutamate synthase	2.88
PSYTB_20306	sodium:alanine symporter	2.87
PSYTB_07336	NADPH:quinone oxidoreductase	2.87
PSYTB_03009	senescence marker protein 30	2.87
PSYTB_24287	HrpL-regulated protein	2.86
PSYTB_09221	iron ABC transporter	2.85
PSYTB_28432	hypothetical protein	2.85
PSYTB_21790	hypothetical protein	2.84
PSYTB_01709	LuxR family transcriptional regulator	2.84
PSYTB_24267	hypothetical protein	2.84
PSYTB_01574	diadenosine tetraphosphatase	2.80
PSYTB_18554	repressor	2.80
PSYTB_02549	mannitol 2-dehydrogenase	2.80
PSYTB_02994	arabinose ABC transporter permease	2.80
PSYTB_25199	chromosome partitioning protein ParA	2.77
PSYTB_08321	acetyl-CoA carboxylase	2.76
PSYTB_18744	hypothetical protein	2.76
PSYTB_28397	acyl carrier protein	2.76
PSYTB_00210	pyridine nucleotide-disulfide oxidoreductase	2.73
PSYTB_12338	NADH:flavin oxidoreductase	2.73

Gene locus	Predicted function	Fold change (Wild type/Δ*psyI* mutant)
PSYTB_16235	maleylacetoacetate isomerase	2.72
PSYTB_00629	feruloyl-CoA synthase	2.71
PSYTB_10718	branched-chain amino acid ABC transporterpermease	2.71
PSYTB_24727	hypothetical protein	2.70
PSYTB_16590	methyl-accepting chemotaxis protein	2.69
PSYTB_21830	hypothetical protein	2.69
PSYTB_03314	gamma-glutamyltransferase	2.69
PSYTB_03249	type VI secretion protein	2.69
PSYTB_23956	ABC transporter permease	2.69
PSYTB_09431	MFS transporter	2.68
PSYTB_12585	D-ribose pyranase	2.68
PSYTB_27442	sugar-binding protein	2.67
PSYTB_28905	hypothetical protein	2.66
PSYTB_07416	methionine ABC transporter substrate-bindingprotein	2.66
PSYTB_08871	ABC transporter ATP-binding protein	2.66
PSYTB_08866	branched-chain amino acid ABC transporterpermease	2.65
PSYTB_16265	homogentisate 1,2-dioxygenase	2.65
PSYTB_10578	succinylglutamate desuccinylase/aspartoacylase	2.63
PSYTB_27742	LysR family transcriptional regulator	2.62
PSYTB_10723	branched-chain amino acid transporter permeasesubunit LivH	2.62
PSYTB_10588	histidine/lysine/arginine/ornithine ABCtransporter permease HisQ	2.61
PSYTB_20261	LysR family transcriptional regulator	2.61
PSYTB_06706	hypothetical protein	2.60
PSYTB_05892	MFS transporter	2.60
PSYTB_13751	isocitrate lyase	2.59
PSYTB_24527	hypothetical protein	2.59
PSYTB_28122	—	2.58
PSYTB_16310	DUF4432 domain-containing protein	2.58
PSYTB_10438	glycerol kinase	2.58
PSYTB_01789	short chain dehydrogenase/reductase	2.57
PSYTB_10803	3-oxoacyl-ACP synthase	2.57
PSYTB_17060	D-galactose 1-dehydrogenase	2.56
PSYTB_06577	cold-shock protein	2.56
PSYTB_23246	addiction module protein	2.55
PSYTB_00589	benzaldehyde dehydrogenase	2.55
PSYTB_23966	membrane protein	2.55
PSYTB_13961	ketoglutarate semialdehyde dehydrogenase	2.55
PSYTB_06557	porin	2.54
PSYTB_00065	hypothetical protein	2.53
PSYTB_21066	hypothetical protein	2.52

Gene locus	Predicted function	Fold change (Wild type/Δ*psyI* mutant)
PSYTB_06896	50S ribosomal protein L33	2.52
PSYTB_21096	GABA permease	2.49
PSYTB_16195	gluconokinase	2.49
PSYTB_04555	LysR family transcriptional regulator	2.48
PSYTB_20916	OprD family outer membrane protein	2.47
PSYTB_18794	cell division protein Fic	2.47
PSYTB_22385	GntR family transcriptional regulator	2.45
PSYTB_10573	histidine ABC transporter ATP-binding protein	2.45
PSYTB_17505	glucarate transporter	2.45
PSYTB_23371	3-dehydroquinate dehydratase	2.44
PSYTB_14650	amidase	2.44
PSYTB_08086	adenosylmethionine--8-amino-7-oxononanoateaminotransferase BioA	2.44
PSYTB_21465	hypothetical protein	2.44
PSYTB_20991	transcriptional regulator	2.43
PSYTB_20541	—	2.43
PSYTB_16580	inositol ABC transporter permease	2.43
PSYTB_19081	RHS repeat-associated core domain-containingprotein	2.42
PSYTB_20706	permease	2.42
PSYTB_00040	hypothetical protein	2.42
PSYTB_06711	hypothetical protein	2.41
PSYTB_00604	3-phenylpropionic acid transporter	2.41
PSYTB_21021	1-(5-phosphoribosyl)-5-((5-phosphoribosylamino)methylideneamino)imidazole-4-carboxamide isomerase	2.41
PSYTB_06976	membrane protein	2.41
PSYTB_07861	glutamate synthase subunit beta	2.41
PSYTB_06981	cyclic nucleotide-binding protein	2.40
PSYTB_19691	LysR family transcriptional regulator	2.40
PSYTB_20221	pyridoxal kinase	2.40
PSYTB_21071	transcriptional regulator	2.40
PSYTB_18434	glutamine amidotransferase	2.39
PSYTB_20426	rubredoxin	2.38
PSYTB_07596	N-formimino-L-glutamate deiminase	2.37
PSYTB_15875	recombination protein RecR	2.37
PSYTB_09656	hypothetical protein	2.36
PSYTB_10788	—	2.36
PSYTB_20666	LacI family transcriptional regulator	2.35
PSYTB_10523	leucyl-tRNA synthetase	2.35
PSYTB_28387	GTP-binding protein	2.35
PSYTB_10793	long-chain-fatty-acid--CoA ligase	2.34
PSYTB_20556	hypothetical protein	2.34

Gene locus	Predicted function	Fold change (Wild type/Δ*psyI* mutant)
PSYTB_20586	cytochrome C	2.33
PSYTB_13410	GntR family transcriptional regulator	2.33
PSYTB_19341	(2Fe-2S)-binding protein	2.33
PSYTB_13435	electron transfer flavoprotein subunit beta	2.32
PSYTB_25009	transcriptional regulator	2.31
PSYTB_23241	addiction module antitoxin	2.31
PSYTB_28482	asparagine synthetase B	2.30
PSYTB_23411	secretin	2.30
PSYTB_06242	30S ribosomal protein S2	2.30
PSYTB_19466	DNA-binding response regulator	2.29
PSYTB_19671	tryptophan synthase subunit beta	2.29
PSYTB_09311	3-ketoacyl-ACP reductase	2.28
PSYTB_22736	preprotein translocase subunit SecY	2.28
PSYTB_23406	hypothetical protein	2.27
PSYTB_06686	GCN5 family acetyltransferase	2.27
PSYTB_16285	hydrolase	2.26
PSYTB_09691	MFS transporter	2.26
PSYTB_08221	membrane protein	2.25
PSYTB_28417	membrane protein	2.24
PSYTB_10733	hypothetical protein	2.24
PSYTB_00759	(2Fe-2S)-binding protein	2.24
PSYTB_17420	flavodoxin	2.24
PSYTB_23441	lipoprotein	2.24
PSYTB_21026	imidazole glycerol phosphate synthase subunitHisH	2.24
PSYTB_05025	D-hexose-6-phosphate mutarotase	2.24
PSYTB_22543	bifunctional biotin--[acetyl-CoA-carboxylase]synthetase/biotin operon repressor	2.23
PSYTB_24904	ATP-dependent DNA helicase Rep	2.22
PSYTB_00290	XylR family transcriptional regulator	2.22
PSYTB_08081	16S rRNA (uracil(1498)-N(3))-methyltransferase	2.22
PSYTB_28052	—	2.21
PSYTB_07176	metal ABC transporter permease	2.20
PSYTB_25284	integrase	2.20
PSYTB_28047	—	2.20
PSYTB_06721	multidrug ABC transporter permease	2.20
PSYTB_18134	alpha/beta hydrolase	2.20
PSYTB_21435	—	2.19
PSYTB_25019	hypothetical protein	2.19
PSYTB_13430	electron transfer flavoprotein subunit beta	2.19
PSYTB_07051	RNA-binding transcriptional accessory protein	2.19
PSYTB_21990	aconitate hydratase B	2.18
PSYTB_28287	(p)ppGpp synthetase	2.18

Gene locus	Predicted function	Fold change (Wild type/Δ*psyI* mutant)
PSYTB_21795	phage shock protein A	2.18
PSYTB_24844	diguanylate cyclase	2.17
PSYTB_14168	glucarate dehydratase	2.17
PSYTB_11493	TonB-dependent receptor	2.17
PSYTB_21440	ADP-ribosylglycohydrolase	2.16
PSYTB_20881	TetR family transcriptional regulator	2.16
PSYTB_26046	glutamyl-Q tRNA(Asp) ligase	2.16
PSYTB_08131	hydroxypyruvate isomerase	2.15
PSYTB_25344	hypothetical protein	2.15
PSYTB_21270	hypothetical protein	2.15
PSYTB_22976	hypothetical protein	2.14
PSYTB_24507	molecular chaperone Hsp33	2.13
PSYTB_28412	hypothetical protein	2.13
PSYTB_24522	hypothetical protein	2.13
PSYTB_25349	integration host factor subunit beta	2.12
PSYTB_20096	nitrate ABC transporter ATP-binding protein	2.12
PSYTB_02169	IclR family transcriptional regulator	2.11
PSYTB_11978	glucose dehydrogenase	2.11
PSYTB_03556	chromosome partitioning protein	2.11
PSYTB_08876	ABC transporter ATP-binding protein	2.11
PSYTB_20651	sulfonate ABC transporter substrate-bindingprotein	2.11
PSYTB_21031	imidazoleglycerol-phosphate dehydratase	2.10
PSYTB_03489	AcrR family transcriptional regulator	2.10
PSYTB_10713	ABC transporter ATP-binding protein	2.10
PSYTB_07741	poly(R)-hydroxyalkanoic acid synthase	2.09
PSYTB_25189	replication protein A	2.09
PSYTB_24859	sulfate/thiosulfate transporter subunit	2.08
PSYTB_05325	AsnC family transcriptional regulator	2.08
PSYTB_19941	glutamine--fructose-6-phosphateaminotransferase	2.08
PSYTB_21016	1-(5-phosphoribosyl)-5-((5-phosphoribosylamino)methylideneamino)imidazole-4-carboxamide isomerase	2.08
PSYTB_00624	salicylaldehyde dehydrogenase	2.08
PSYTB_08576	phosphomethylpyrimidine synthase ThiC	2.08
PSYTB_13986	TetR family transcriptional regulator	2.08
PSYTB_23396	type II secretion system protein F	2.08
PSYTB_21695	alkanesulfonate monooxygenase	2.07
PSYTB_21036	aromatic amino acid aminotransferase	2.07
PSYTB_03499	histidine kinase	2.05
PSYTB_20286	GntR family transcriptional regulator	2.04
PSYTB_15280	AraC family transcriptional regulator	2.03
PSYTB_21260	agmatine deiminase	2.03

Gene locus	Predicted function	Fold change (Wild type/Δ*psyI* mutant)
PSYTB_19696	choline-sulfatase	2.02
PSYTB_07551	D-cysteine desulfhydrase	2.01
PSYTB_19096	acyltransferase	2.01
PSYTB_19621	aminopeptidase	2.01
PSYTB_20301	L-asparaginase 1	2.00
PSYTB_26281	recombination-associated protein RdgC	2.00
PSYTB_17535	diguanylate cyclase	2.00
PSYTB_10983	hypothetical protein	2.00
PSYTB_07546	cystine transporter subunit	2.00
PSYTB_22180	arginine succinyltransferase	-2.00
PSYTB_02629	hypothetical protein	-2.01
PSYTB_07366	cell division protein ZapA	-2.01
PSYTB_11818	LacI family transcriptional regulator	-2.01
PSYTB_21410	type VI secretion system protein ImpM	-2.01
PSYTB_05767	hypothetical protein	-2.01
PSYTB_18269	cell division protein FtsA	-2.01
PSYTB_13641	peptidase	-2.02
PSYTB_26186	peptidase S41	-2.02
PSYTB_18284	hypothetical protein	-2.02
PSYTB_15030	glucoamylase	-2.02
PSYTB_25861	phosphoribosyltransferase	-2.02
PSYTB_16460	hypothetical protein	-2.03
PSYTB_22035	hypothetical protein	-2.03
PSYTB_17520	membrane protein	-2.03
PSYTB_12935	3-methylitaconate isomerase	-2.03
PSYTB_13505	diguanylate cyclase	-2.03
PSYTB_22175	arginine N-succinyltransferase	-2.04
PSYTB_16500	cytochrome B561	-2.04
PSYTB_13761	secretin	-2.04
PSYTB_25419	membrane protein	-2.04
PSYTB_17560	methyl-accepting chemotaxis protein	-2.04
PSYTB_08391	amino acid ABC transporter substrate-bindingprotein	-2.05
PSYTB_00679	gluconolaconase	-2.05
PSYTB_24142	MFS transporter	-2.05
PSYTB_08171	membrane protein	-2.05
PSYTB_21255	—	-2.06
PSYTB_03616	phosphatidylcholine synthase	-2.06
PSYTB_28665	hypothetical protein	-2.06
PSYTB_05180	acetyltransferase	-2.06
PSYTB_10828	3-oxoadipate enol-lactonase	-2.06
PSYTB_16185	carbon storage regulator	-2.06
PSYTB_16020	molybdopterin-binding oxidoreductase	-2.07

Gene locus	Predicted function	Fold change (Wild type/Δ*psyI* mutant)
PSYTB_27882	type III effector	-2.07
PSYTB_12078	—	-2.07
PSYTB_16015	thioredoxin reductase	-2.07
PSYTB_23086	hypothetical protein	-2.07
PSYTB_19276	HDOD domain-containing protein	-2.07
PSYTB_15385	cyanate hydratase	-2.07
PSYTB_15505	sigma-54-dependent Fis family transcriptionalregulator	-2.08
PSYTB_18876	acyl-CoA dehydrogenase	-2.08
PSYTB_26211	peptidyl-prolyl cis-trans isomerase	-2.08
PSYTB_16750	lysine transporter LysE	-2.08
PSYTB_24147	DSBA oxidoreductase	-2.09
PSYTB_25464	membrane protein	-2.09
PSYTB_26366	copper resistance protein B	-2.09
PSYTB_27352	membrane protein	-2.09
PSYTB_01104	epimerase	-2.09
PSYTB_06497	RNA-binding protein S4	-2.10
PSYTB_22030	two-component sensor histidine kinase	-2.10
PSYTB_11538	ABC transporter	-2.10
PSYTB_26650	multidrug ABC transporter substrate-bindingprotein	-2.10
PSYTB_04018	hypothetical protein	-2.11
PSYTB_05415	—	-2.11
PSYTB_17480	sensor histidine kinase	-2.11
PSYTB_11543	serine protein kinase PrkA	-2.11
PSYTB_11433	peptide deformylase	-2.11
PSYTB_18009	phosphocarrier protein HPr	-2.11
PSYTB_13315	dienelactone hydrolase	-2.11
PSYTB_00475	molybdate ABC transporter substrate-bindingprotein	-2.12
PSYTB_21575	hypothetical protein	-2.12
PSYTB_18821	coenzyme PQQ biosynthesis protein PqqF	-2.12
PSYTB_27052	hypothetical protein	-2.12
PSYTB_11378	RNA helicase	-2.12
PSYTB_05160	cytochrome O ubiquinol oxidase	-2.13
PSYTB_20671	beta-aspartyl-peptidase	-2.13
PSYTB_07816	pilus assembly protein PilN	-2.13
PSYTB_17335	hypothetical protein	-2.13
PSYTB_17815	dihydropteroate synthase	-2.13
PSYTB_14373	DNA-binding response regulator	-2.13
PSYTB_10378	5-methyltetrahydropteroyltriglutamate--homocysteine methyltransferase	-2.14
PSYTB_02404	sugar ABC transporter substrate-binding protein	-2.14
PSYTB_15050	hypothetical protein	-2.14
PSYTB_19001	hypothetical protein	-2.14

Gene locus	Predicted function	Fold change (Wild type/Δ*psyI* mutant)
PSYTB_01099	haloacid dehalogenase	-2.14
PSYTB_28592	—	-2.14
PSYTB_13816	ATP-dependent Clp protease adaptor ClpS	-2.15
PSYTB_04075	hypothetical protein	-2.15
PSYTB_22415	peptidase M20	-2.15
PSYTB_11618	ABC transporter permease	-2.15
PSYTB_22911	ABC transporter substrate-binding protein	-2.15
PSYTB_21620	hybrid sensor histidine kinase/responseregulator	-2.15
PSYTB_21785	aminopeptidase N	-2.16
PSYTB_06951	hypothetical protein	-2.16
PSYTB_12765	acyltransferase	-2.17
PSYTB_21580	RNA helicase	-2.17
PSYTB_11798	transporter	-2.17
PSYTB_04840	glycine cleavage system protein H	-2.17
PSYTB_13065	hypothetical protein	-2.17
PSYTB_00964	hypothetical protein	-2.17
PSYTB_12043	phosphonate ABC transporter	-2.17
PSYTB_26361	copper oxidase	-2.17
PSYTB_22040	DNA-binding response regulator	-2.18
PSYTB_17345	hypothetical protein	-2.18
PSYTB_10758	hypothetical protein	-2.18
PSYTB_13120	hypothetical protein	-2.18
PSYTB_04013	outer membrane lipoprotein LolB	-2.18
PSYTB_15925	thiol:disulfide interchange protein	-2.18
PSYTB_10638	hypothetical protein	-2.19
PSYTB_11453	tRNA-specific adenosine deaminase	-2.19
PSYTB_28815	hypothetical protein	-2.19
PSYTB_05420	hypothetical protein	-2.19
PSYTB_03883	—	-2.20
PSYTB_11483	glucose/sorbosone family dehydrogenase	-2.20
PSYTB_00969	hypothetical protein	-2.20
PSYTB_09866	DNA helicase UvrD	-2.20
PSYTB_04485	hypothetical protein	-2.21
PSYTB_25394	bifunctional 3-demethylubiquinone3-O-methyltransferase/2-octaprenyl-6-hydroxy phenolmethylase	-2.21
PSYTB_07821	pilus assembly protein PilP	-2.21
PSYTB_17040	hypothetical protein	-2.21
PSYTB_16465	hypothetical protein	-2.21
PSYTB_05140	cytochrome ubiquinol oxidase subunit II	-2.21
PSYTB_27222	ethyl tert-butyl ether degradation protein EthD	-2.22
PSYTB_27037	GntR family transcriptional regulator	-2.23

Gene locus	Predicted function	Fold change (Wild type/Δ*psyI* mutant)
PSYTB_07831	fimbrial protein	-2.23
PSYTB_07231	polyphosphate kinase	-2.24
PSYTB_11633	hypothetical protein	-2.24
PSYTB_25324	membrane protein	-2.24
PSYTB_07566	DNA-directed RNA polymerase sigma-70 factor	-2.24
PSYTB_16355	glycosyl transferase family 1	-2.24
PSYTB_15350	pilus assembly protein PilZ	-2.25
PSYTB_11478	hypothetical protein	-2.25
PSYTB_02159	ketodeoxygluconokinase	-2.25
PSYTB_02514	glycosyltransferase	-2.25
PSYTB_19271	formamidopyrimidine-DNA glycosylase	-2.26
PSYTB_02799	malate:quinone oxidoreductase	-2.26
PSYTB_17979	peptidase C69	-2.26
PSYTB_26977	exonuclease	-2.27
PSYTB_12073	methyl-accepting chemotaxis protein	-2.27
PSYTB_12013	carbon-phosphorus lyase complex subunit PhnI	-2.27
PSYTB_21086	pili assembly chaperone	-2.28
PSYTB_16130	hypothetical protein	-2.28
PSYTB_03771	transcriptional regulator	-2.29
PSYTB_04080	amine oxidase	-2.29
PSYTB_23486	DNA-binding response regulator	-2.29
PSYTB_21136	molecular chaperone DnaJ	-2.30
PSYTB_02234	heme ABC transporter ATP-binding protein	-2.30
PSYTB_27662	xanthine dehydrogenase	-2.30
PSYTB_21495	—	-2.30
PSYTB_17640	diguanylate cyclase	-2.30
PSYTB_20731	hypothetical protein	-2.30
PSYTB_14725	hypothetical protein	-2.30
PSYTB_12975	alpha-L-glutamate ligase	-2.31
PSYTB_22265	hypothetical protein	-2.31
PSYTB_22936	arylesterase	-2.31
PSYTB_20076	hypothetical protein	-2.31
PSYTB_15880	hypothetical protein	-2.31
PSYTB_25826	—	-2.31
PSYTB_23496	pilus assembly protein TadE	-2.32
PSYTB_25761	chemotaxis protein	-2.32
PSYTB_04905	multidrug transporter	-2.32
PSYTB_13110	transglutaminase	-2.32
PSYTB_15020	glucose-6-phosphate 1-dehydrogenase	-2.32
PSYTB_26206	hypothetical protein	-2.32
PSYTB_24112	ACP phosphodiesterase	-2.33
PSYTB_24167	—	-2.33

Gene locus	Predicted function	Fold change (Wild type/Δ*psyI* mutant)
PSYTB_08771	exoribonuclease R	-2.33
PSYTB_19526	DNA-binding response regulator	-2.33
PSYTB_12113	membrane protein	-2.33
PSYTB_09231	calcium:proton antiporter	-2.34
PSYTB_25469	DTW domain-containing protein	-2.34
PSYTB_17525	histidine kinase	-2.35
PSYTB_05355	peptide transporter	-2.35
PSYTB_02199	2-oxoacid:ferredoxin oxidoreductase	-2.35
PSYTB_00684	antibiotic biosynthesis monooxygenase	-2.36
PSYTB_15805	chemotaxis protein	-2.36
PSYTB_14413	chemotaxis protein	-2.37
PSYTB_19656	hypothetical protein	-2.37
PSYTB_25454	class II fumarate hydratase	-2.37
PSYTB_27002	NADP-dependent oxidoreductase	-2.38
PSYTB_21420	type VI secretion system effector	-2.38
PSYTB_09661	DSBA oxidoreductase	-2.38
PSYTB_12238	chemotaxis protein	-2.38
PSYTB_07626	glycogen phosphorylase	-2.39
PSYTB_18826	pyrroloquinoline quinone biosynthesis protein B	-2.39
PSYTB_27777	non-ribosomal peptide synthetase	-2.39
PSYTB_29390	—	-2.40
PSYTB_12655	hypothetical protein	-2.40
PSYTB_03851	hypothetical protein	-2.40
PSYTB_15485	flagellar protein FlaG	-2.40
PSYTB_01334	hypothetical protein	-2.41
PSYTB_28222	C4-dicarboxylate ABC transporter	-2.41
PSYTB_12398	SDR family oxidoreductase	-2.41
PSYTB_11388	hypothetical protein	-2.42
PSYTB_10388	DSBA oxidoreductase	-2.42
PSYTB_16360	glycosyl transferase	-2.42
PSYTB_28760	hypothetical protein	-2.42
PSYTB_12083	histidine kinase	-2.42
PSYTB_02114	hypothetical protein	-2.43
PSYTB_26590	3-ketoacyl-ACP reductase	-2.43
PSYTB_27172	hypothetical protein	-2.43
PSYTB_27782	—	-2.44
PSYTB_12233	chemotaxis protein CheW	-2.44
PSYTB_03761	diguanylate cyclase	-2.44
PSYTB_11083	—	-2.44
PSYTB_12825	23S rRNA methyltransferase	-2.44
PSYTB_17515	hypothetical protein	-2.45
PSYTB_17195	metallopeptidase	-2.45

Gene locus	Predicted function	Fold change (Wild type/Δ*psyI* mutant)
PSYTB_09306	hypothetical protein	-2.45
PSYTB_02509	transcriptional regulator	-2.45
PSYTB_07811	pilus assembly protein PilM	-2.45
PSYTB_05145	cytochrome ubiquinol oxidase subunit I	-2.45
PSYTB_02259	alpha-amylase	-2.46
PSYTB_04635	alginate biosynthesis protein AlgK	-2.46
PSYTB_17490	ABC transporter ATP-binding protein	-2.46
PSYTB_18836	coenzyme PQQ synthesis protein D	-2.46
PSYTB_02764	succinate dehydrogenase	-2.47
PSYTB_06012	hybrid sensor histidine kinase/responseregulator	-2.47
PSYTB_21425	type VI secretion protein ImpA	-2.47
PSYTB_19451	—	-2.48
PSYTB_06027	chemotaxis protein	-2.48
PSYTB_24467	adenylate cyclase	-2.48
PSYTB_27717	membrane protein	-2.48
PSYTB_00185	sigma-54-dependent Fis family transcriptionalregulator	-2.48
PSYTB_01964	phosphodiesterase	-2.49
PSYTB_04420	hypothetical protein	-2.50
PSYTB_17175	GNAT family acetyltransferase	-2.50
PSYTB_21485	—	-2.50
PSYTB_01744	LuxR family transcriptional regulator	-2.51
PSYTB_11403	hypothetical protein	-2.51
PSYTB_03756	chemotaxis protein CheC	-2.51
PSYTB_15345	MFS transporter	-2.51
PSYTB_06032	chemotaxis protein CheW	-2.52
PSYTB_09821	hypothetical protein	-2.52
PSYTB_12670	rhamnosyltransferase	-2.53
PSYTB_07006	ribonucleotide reductase	-2.53
PSYTB_27072	hypothetical protein	-2.53
PSYTB_24914	MATE family efflux transporter	-2.54
PSYTB_17969	protease TldD	-2.54
PSYTB_01109	oxidoreductase	-2.54
PSYTB_12558	hypothetical protein	-2.54
PSYTB_02624	MFS transporter	-2.54
PSYTB_24172	chemotaxis protein	-2.55
PSYTB_16340	membrane protein	-2.55
PSYTB_09476	cardiolipin synthase B	-2.55
PSYTB_28780	hypothetical protein	-2.55
PSYTB_11998	phosphonate ABC transporter ATP-binding protein	-2.55
PSYTB_05972	histidine kinase	-2.55
PSYTB_04505	ribosomal subunit interface protein	-2.55
PSYTB_23476	hypothetical protein	-2.57

Gene locus	Predicted function	Fold change (Wild type/Δ*psyI* mutant)
PSYTB_14705	hypothetical protein	-2.58
PSYTB_14715	paraquat-inducible protein A	-2.59
PSYTB_21490	hypothetical protein	-2.59
PSYTB_12970	membrane protein	-2.59
PSYTB_12008	carbon-phosphorus lyase complex subunit PhnJ	-2.59
PSYTB_16955	apolipoprotein N-acyltransferase	-2.59
PSYTB_26752	hypothetical protein	-2.59
PSYTB_25069	copper resistance protein C	-2.60
PSYTB_22365	hypothetical protein	-2.60
PSYTB_26066	(2Fe-2S)-binding protein	-2.60
PSYTB_16375	LPS biosynthesis protein	-2.60
PSYTB_02419	oxidoreductase	-2.60
PSYTB_13100	acyl-CoA thioesterase	-2.60
PSYTB_04070	cyclopropane-fatty-acyl-phospholipid synthase	-2.61
PSYTB_21415	membrane protein	-2.61
PSYTB_19641	peroxiredoxin	-2.61
PSYTB_01719	—	-2.61
PSYTB_17900	4-carboxymuconolactone decarboxylase	-2.61
PSYTB_04110	hypothetical protein	-2.62
PSYTB_16370	glycosyl transferase family 1	-2.63
PSYTB_13821	ATP-dependent Clp protease ATP-binding subunitClpA	-2.63
PSYTB_04910	RND transporter	-2.63
PSYTB_27672	guanine deaminase	-2.64
PSYTB_04065	membrane protein	-2.65
PSYTB_00799	hybrid sensor histidine kinase/responseregulator	-2.65
PSYTB_16380	sugar ABC transporter substrate-binding protein	-2.65
PSYTB_22045	translation initiation factor 2	-2.66
PSYTB_15355	flagellar synthesis chaperone protein FlgN	-2.66
PSYTB_25459	NAD(P)H dehydrogenase	-2.68
PSYTB_20336	transglycosylase	-2.68
PSYTB_02109	aldo/keto reductase	-2.68
PSYTB_07286	calcium transporter ChaC	-2.69
PSYTB_25409	hypothetical protein	-2.69
PSYTB_22776	bacterioferritin	-2.70
PSYTB_10383	alpha/beta hydrolase	-2.70
PSYTB_04835	glycine dehydrogenase (decarboxylating)	-2.70
PSYTB_15025	hypothetical protein	-2.73
PSYTB_15885	flagellar hook-length control protein FliK	-2.74
PSYTB_22941	mechanosensitive ion channel protein	-2.74
PSYTB_18841	pyrroloquinoline quinone biosynthesis proteinPqqE	-2.74
PSYTB_12088	endonuclease	-2.75
PSYTB_23111	transcriptional regulator	-2.75

Gene locus	Predicted function	Fold change (Wild type/Δ*psyI* mutant)
PSYTB_11993	phosphonate metabolism protein PhnM	-2.75
PSYTB_01394	sensory protein TspO	-2.76
PSYTB_27667	xanthine dehydrogenase accessory factor XdhC	-2.76
PSYTB_15560	fused response regulator/phosphatase	-2.76
PSYTB_27332	membrane protein	-2.76
PSYTB_18831	pyrroloquinoline quinone biosynthesis protein C	-2.77
PSYTB_02829	transcriptional regulator	-2.78
PSYTB_12003	phosphonate C-P lyase	-2.79
PSYTB_01074	oxidoreductase	-2.80
PSYTB_01314	type VI secretion protein ImpB	-2.80
PSYTB_14955	glycosyl transferase	-2.80
PSYTB_14023	general secretion pathway protein GspH	-2.80
PSYTB_03631	—	-2.81
PSYTB_01729	—	-2.81
PSYTB_25751	sulfate transporter	-2.82
PSYTB_14720	paraquat-inducible protein A	-2.82
PSYTB_15480	flagellin	-2.82
PSYTB_16390	capsular polysaccharide biosynthesis protein	-2.82
PSYTB_27127	helix-turn-helix transcriptional regulator	-2.84
PSYTB_04920	transcriptional regulator	-2.85
PSYTB_10243	hypothetical protein	-2.85
PSYTB_14138	formate transporter	-2.86
PSYTB_18846	peptidase S9	-2.86
PSYTB_16420	lytic transglycosylase	-2.86
PSYTB_13270	alpha/beta hydrolase	-2.87
PSYTB_17880	hypothetical protein	-2.87
PSYTB_04590	mannose-1-phosphate guanylyltransferase	-2.87
PSYTB_29075	—	-2.88
PSYTB_05220	superoxide dismutase	-2.88
PSYTB_02479	hybrid sensor histidine kinase/responseregulator	-2.89
PSYTB_26767	—	-2.89
PSYTB_00729	—	-2.89
PSYTB_14965	acetylglucosaminylphosphatidylinositoldeacetylase	-2.90
PSYTB_11733	ribonucleotide-diphosphate reductase subunitalpha	-2.90
PSYTB_10248	hypothetical protein	-2.90
PSYTB_01724	—	-2.90
PSYTB_20171	hypothetical protein	-2.91
PSYTB_21220	hypothetical protein	-2.92
PSYTB_16320	hypothetical protein	-2.93
PSYTB_25474	hypothetical protein	-2.93
PSYTB_07601	lipocalin	-2.93
PSYTB_29570	—	-2.93

Gene locus	Predicted function	Fold change (Wild type/Δ*psyI* mutant)
PSYTB_14790	hypothetical protein	-2.94
PSYTB_16385	mannose-1-phosphate guanylyltransferase	-2.94
PSYTB_23126	phosphate starvation-inducible protein PsiF	-2.95
PSYTB_13881	glutathione-dependent reductase	-2.95
PSYTB_04915	two-component system response regulator	-2.96
PSYTB_10738	diguanylate phosphodiesterase	-2.96
PSYTB_01734	—	-2.97
PSYTB_19091	hypothetical protein	-2.97
PSYTB_05410	two-component system sensor histidine kinase	-2.97
PSYTB_15055	hypothetical protein	-2.98
PSYTB_19011	methyl-accepting chemotaxis protein	-2.99
PSYTB_28695	glycerol-3-phosphate dehydrogenase	-2.99
PSYTB_07216	glutamine amidotransferase	-2.99
PSYTB_10238	hypothetical protein	-2.99
PSYTB_27147	membrane protein	-2.99
PSYTB_17390	glycine/betaine ABC transporter ATP-bindingprotein	-3.00
PSYTB_02669	oxidoreductase	-3.00
PSYTB_06956	alanine racemase	-3.01
PSYTB_03626	chemotaxis protein	-3.02
PSYTB_17385	choline ABC transporter permease	-3.02
PSYTB_16365	beta-xylosidase	-3.02
PSYTB_26006	glucose-6-phosphate isomerase	-3.03
PSYTB_23881	—	-3.03
PSYTB_27752	—	-3.04
PSYTB_14710	mammalian cell entry protein	-3.04
PSYTB_04320	membrane protein	-3.04
PSYTB_21895	hypothetical protein	-3.04
PSYTB_15375	chemotaxis protein CheR	-3.05
PSYTB_16175	hypothetical protein	-3.05
PSYTB_26001	histidine kinase	-3.05
PSYTB_00724	LysR family transcriptional regulator	-3.05
PSYTB_13075	lytic transglycosylase F	-3.06
PSYTB_17015	sensor histidine kinase	-3.07
PSYTB_23886	AraC family transcriptional regulator	-3.07
PSYTB_11488	peptidase M4	-3.07
PSYTB_14730	peptidase	-3.08
PSYTB_26987	hypothetical protein	-3.08
PSYTB_17215	(2Fe-2S)-binding protein	-3.08
PSYTB_00784	glycosyl transferase	-3.09
PSYTB_16140	methyl-accepting chemotaxis protein	-3.09
PSYTB_03651	GDP-6-deoxy-D-lyxo-4-hexulose reductase	-3.09
PSYTB_14001	type II secretion system protein GspE	-3.09

Gene locus	Predicted function	Fold change (Wild type/Δ*psyI* mutant)
PSYTB_24874	membrane protein	-3.10
PSYTB_09116	acetyl-CoA acetyltransferase	-3.11
PSYTB_25484	hypothetical protein	-3.12
PSYTB_02574	zinc-binding dehydrogenase	-3.12
PSYTB_11728	ribonucleotide-diphosphate reductase subunitbeta	-3.13
PSYTB_17045	hypothetical protein	-3.13
PSYTB_04935	acetyl-CoA carboxylase	-3.13
PSYTB_09111	3-ketoacyl-ACP reductase	-3.14
PSYTB_27197	—	-3.15
PSYTB_28352	polyketide cyclase	-3.16
PSYTB_22240	DNA-binding protein	-3.16
PSYTB_14418	—	-3.16
PSYTB_15415	flagellar hook protein FlgE	-3.18
PSYTB_20216	hypothetical protein	-3.18
PSYTB_27367	—	-3.18
PSYTB_01159	hypothetical protein	-3.19
PSYTB_02769	flavin reductase	-3.21
PSYTB_16345	acetyltransferase	-3.21
PSYTB_25746	chemotaxis protein CheA	-3.21
PSYTB_18694	hypothetical protein	-3.21
PSYTB_14980	hypothetical protein	-3.22
PSYTB_12048	chemotaxis protein	-3.22
PSYTB_26672	chromosome segregation ATPase	-3.22
PSYTB_27152	hypothetical protein	-3.22
PSYTB_01499	diguanylate phosphodiesterase	-3.22
PSYTB_11533	polyribonucleotide nucleotidyltransferase(polynucleotide phosphorylase)	-3.23
PSYTB_15700	chemotaxis protein	-3.24
PSYTB_28392	chemotaxis sensory transducer	-3.26
PSYTB_02584	NAD dependent epimerase/dehydratase	-3.26
PSYTB_18851	hypothetical protein	-3.26
PSYTB_12690	hypothetical protein	-3.27
PSYTB_08676	flagellar motor protein MotA	-3.28
PSYTB_17375	glycine/betaine ABC transporter permease	-3.28
PSYTB_27292	hypothetical protein	-3.29
PSYTB_23501	histidine kinase	-3.30
PSYTB_25741	—	-3.30
PSYTB_16675	ATP-dependent DNA ligase	-3.31
PSYTB_09816	hypothetical protein	-3.32
PSYTB_15650	chemotaxis protein CheA	-3.32
PSYTB_09526	pilus assembly protein PilZ	-3.32
PSYTB_16410	chemotaxis protein CheW	-3.33

Gene locus	Predicted function	Fold change (Wild type/Δ*psyI* mutant)
PSYTB_24107	hypothetical protein	-3.33
PSYTB_02204	hemerythrin	-3.34
PSYTB_16255	diguanylate cyclase	-3.34
PSYTB_23471	NUDIX hydrolase	-3.35
PSYTB_10518	DNA-binding response regulator	-3.37
PSYTB_24834	diguanylate cyclase	-3.37
PSYTB_14735	—	-3.38
PSYTB_27772	taurine dioxygenase	-3.39
PSYTB_16125	chemotaxis protein	-3.39
PSYTB_10158	RNA polymerase sigma factor AlgU	-3.41
PSYTB_21380	type VI secretion system protein	-3.41
PSYTB_17330	membrane protein	-3.41
PSYTB_03836	—	-3.42
PSYTB_00734	ATPase	-3.42
PSYTB_17380	glycine/betaine ABC transportersubstrate-binding protein	-3.42
PSYTB_27252	hypothetical protein	-3.42
PSYTB_13105	haloacid dehalogenase	-3.43
PSYTB_15690	chemotaxis protein	-3.43
PSYTB_05245	diguanylate cyclase	-3.44
PSYTB_00315	methyl-accepting chemotaxis protein	-3.45
PSYTB_10428	—	-3.46
PSYTB_10228	lon protease	-3.46
PSYTB_27137	hypothetical protein	-3.46
PSYTB_00999	succinate dehydrogenase	-3.46
PSYTB_25756	two-component system response regulator	-3.47
PSYTB_32117	—	-3.47
PSYTB_10448	aminoacyl-tRNA deacylase	-3.48
PSYTB_27512	hypothetical protein	-3.48
PSYTB_29485	—	-3.49
PSYTB_11428	hypothetical protein	-3.50
PSYTB_15370	chemotaxis protein CheW	-3.51
PSYTB_17485	two-component system response regulator	-3.52
PSYTB_16305	dimethylallyltransferase	-3.52
PSYTB_22245	hypothetical protein	-3.53
PSYTB_20081	chemotaxis protein	-3.53
PSYTB_12053	histidine kinase	-3.53
PSYTB_08681	flagellar motor protein MotB	-3.54
PSYTB_10568	—	-3.54
PSYTB_14013	type II secretion system protein GspF	-3.56
PSYTB_15555	anti-anti-sigma regulatory factor	-3.57
PSYTB_15670	flagellar motor protein MotD	-3.58

Gene locus	Predicted function	Fold change (Wild type/Δ*psyI* mutant)
PSYTB_26311	hypothetical protein	-3.59
PSYTB_14018	type II secretion system protein GspG	-3.60
PSYTB_22395	glutathione-dependent formaldehydedehydrogenase	-3.61
PSYTB_15665	flagellar motor protein	-3.62
PSYTB_24817	NAD-dependent succinate-semialdehydedehydrogenase	-3.63
PSYTB_15680	chemotaxis protein CheW	-3.63
PSYTB_03656	chemotaxis protein	-3.64
PSYTB_28735	—	-3.64
PSYTB_12148	MFS transporter	-3.67
PSYTB_27527	hypothetical protein	-3.68
PSYTB_17230	cytochrome D ubiquinol oxidase subunit I	-3.68
PSYTB_26757	universal stress protein	-3.69
PSYTB_02019	diguanylate cyclase	-3.72
PSYTB_02959	amino acid permease	-3.72
PSYTB_22956	outer membrane protein W	-3.75
PSYTB_10011	hypothetical protein	-3.76
PSYTB_16505	endonuclease	-3.76
PSYTB_02954	hypothetical protein	-3.76
PSYTB_15000	glycogen synthase	-3.77
PSYTB_18699	SpoVR family protein	-3.82
PSYTB_25941	—	-3.82
PSYTB_15940	hypothetical protein	-3.83
PSYTB_12228	—	-3.83
PSYTB_19661	hypothetical protein	-3.83
PSYTB_15675	cobyric acid synthase	-3.84
PSYTB_15645	protein phosphatase	-3.85
PSYTB_16945	hypothetical protein	-3.89
PSYTB_17225	aldehyde oxidase	-3.90
PSYTB_04650	GDP-mannose 6-dehydrogenase	-3.90
PSYTB_04925	PAS domain-containing sensor histidine kinase	-3.90
PSYTB_10598	ATP-dependent DNA ligase	-3.91
PSYTB_20086	methyl-accepting chemotaxis protein	-3.92
PSYTB_02604	hypothetical protein	-3.93
PSYTB_27592	glucoamylase	-3.95
PSYTB_11443	histidine ABC transporter substrate-bindingprotein	-3.96
PSYTB_18414	transcriptional regulator	-3.97
PSYTB_14985	malto-oligosyltrehalose synthase	-3.97
PSYTB_04640	hemolysin D	-3.99
PSYTB_04830	glycine cleavage system protein T	-3.99
PSYTB_15640	chemotaxis protein CheY	-4.00
PSYTB_26742	glucan biosynthesis protein D	-4.01
PSYTB_26902	hypothetical protein	-4.01

Gene locus	Predicted function	Fold change (Wild type/Δ*psyI* mutant)
PSYTB_10563	DNA ligase-associated DEXH box helicase	-4.01
PSYTB_13536	excinuclease ABC subunit B	-4.02
PSYTB_15685	chemotaxis protein CheW	-4.02
PSYTB_10603	DNA ligase-associated DEXH box helicase	-4.02
PSYTB_10753	hypothetical protein	-4.03
PSYTB_25559	sorbosone dehydrogenase	-4.03
PSYTB_23481	diguanylate cyclase	-4.03
PSYTB_26376	hypothetical protein	-4.03
PSYTB_06017	hypothetical protein	-4.04
PSYTB_15010	hypothetical protein	-4.06
PSYTB_02779	hypothetical protein	-4.06
PSYTB_15455	flagellar hook-associated protein FlgL	-4.07
PSYTB_10498	membrane protein	-4.07
PSYTB_25766	pyridine nucleotide-disulfide oxidoreductase	-4.07
PSYTB_21375	type VI secretion system protein ImpG	-4.11
PSYTB_02474	circadian clock protein KaiC	-4.12
PSYTB_23116	hypothetical protein	-4.14
PSYTB_25711	chemotaxis response regulator protein-glutamatemethylesterase	-4.14
PSYTB_14970	acyl-CoA dehydrogenase	-4.14
PSYTB_15495	flagellar protein FliS	-4.14
PSYTB_03841	methyl-accepting chemotaxis protein	-4.15
PSYTB_17325	hypothetical protein	-4.15
PSYTB_15550	flagellar protein FliJ	-4.18
PSYTB_24707	hypothetical protein	-4.20
PSYTB_01319	methyl-accepting chemotaxis protein	-4.21
PSYTB_03646	GDP-mannose 4,6-dehydratase	-4.23
PSYTB_01504	chemotaxis protein	-4.24
PSYTB_15635	—	-4.25
PSYTB_17320	elongation factor GreAB	-4.26
PSYTB_24162	hypothetical protein	-4.26
PSYTB_20176	N-acetylmuramoyl-L-alanine amidase	-4.26
PSYTB_15630	DNA-directed RNA polymerase sigma-70 factor	-4.27
PSYTB_00744	histidine kinase	-4.31
PSYTB_03641	diguanylate cyclase	-4.37
PSYTB_09161	hypothetical protein	-4.37
PSYTB_24722	haloacid dehalogenase	-4.38
PSYTB_28810	—	-4.38
PSYTB_02564	hypothetical protein	-4.40
PSYTB_22250	hypothetical protein	-4.40
PSYTB_10478	bacterioferritin	-4.42
PSYTB_15500	flagellar assembly protein FliT	-4.45

Gene locus	Predicted function	Fold change (Wild type/Δ*psyI* mutant)
PSYTB_26762	two-component system response regulator	-4.47
PSYTB_05130	chemotaxis protein CheW	-4.48
PSYTB_20046	SAM-dependent methyltransferase	-4.51
PSYTB_10163	sigma factor AlgU negative regulatory protein	-4.52
PSYTB_15570	flagellar hook-length control protein	-4.52
PSYTB_25576	—	-4.53
PSYTB_14950	dienelactone hydrolase	-4.53
PSYTB_16395	lipid kinase	-4.54
PSYTB_24732	hypothetical protein	-4.54
PSYTB_25736	chemotaxis protein	-4.55
PSYTB_05350	glutathione S-transferase	-4.57
PSYTB_25726	chemotaxis protein CheW	-4.58
PSYTB_10548	cold-shock protein CapB	-4.59
PSYTB_14995	malto-oligosyltrehalose trehalohydrolase	-4.59
PSYTB_25721	SAM-dependent methyltransferase	-4.59
PSYTB_11383	hypothetical protein	-4.59
PSYTB_06147	hypothetical protein	-4.61
PSYTB_17100	hypothetical protein	-4.62
PSYTB_17220	FAD-binding molybdopterin dehydrogenase	-4.64
PSYTB_10328	glycosyl transferase family 51	-4.64
PSYTB_02264	alpha-1,4-glucan--maltose-1-phosphatemaltosyltransferase	-4.64
PSYTB_17720	hypothetical protein	-4.64
PSYTB_10233	hypothetical protein	-4.64
PSYTB_05982	hypothetical protein	-4.65
PSYTB_02484	hypothetical protein	-4.65
PSYTB_06007	oxidoreductase	-4.65
PSYTB_15990	alginate lyase	-4.65
PSYTB_15450	—	-4.69
PSYTB_24884	—	-4.69
PSYTB_14108	ACR family transporter	-4.70
PSYTB_27757	chemotaxis protein	-4.70
PSYTB_21355	hypothetical protein	-4.70
PSYTB_02219	hypothetical protein	-4.72
PSYTB_12775	membrane protein	-4.73
PSYTB_23506	—	-4.73
PSYTB_26416	gas vesicle protein	-4.74
PSYTB_12780	hypothetical protein	-4.76
PSYTB_05125	GNAT family acetyltransferase	-4.76
PSYTB_15610	flagellar biosynthesis protein FlhB	-4.77
PSYTB_09521	hypothetical protein	-4.77
PSYTB_25716	chemotaxis protein CheD	-4.77

Gene locus	Predicted function	Fold change (Wild type/Δ*psyI* mutant)
PSYTB_01739	non-ribosomal peptide synthetase	-4.81
PSYTB_15545	ATP synthase	-4.84
PSYTB_03831	3-oxoacyl-ACP reductase	-4.84
PSYTB_26076	hypothetical protein	-4.84
PSYTB_23811	hypothetical protein	-4.85
PSYTB_01419	—	-4.87
PSYTB_14975	glycogen debranching enzyme	-4.90
PSYTB_15605	flagellar biosynthesis protein FliR	-4.93
PSYTB_06097	membrane protein	-4.96
PSYTB_02784	aldehyde oxidase	-4.97
PSYTB_13085	chemotaxis protein	-5.00
PSYTB_21365	type VI secretion protein EvpB	-5.02
PSYTB_16515	membrane protein	-5.04
PSYTB_12770	membrane protein	-5.04
PSYTB_21370	type VI secretion protein	-5.05
PSYTB_29230	hypothetical protein	-5.06
PSYTB_24737	transcriptional regulator	-5.06
PSYTB_02794	xanthine dehydrogenase	-5.07
PSYTB_06102	chromosome segregation ATPase	-5.11
PSYTB_27597	glucose-1-dehydrogenase	-5.12
PSYTB_02789	hypothetical protein	-5.14
PSYTB_24697	class V aminotransferase	-5.19
PSYTB_14485	hypothetical protein	-5.23
PSYTB_04500	hypothetical protein	-5.27
PSYTB_10168	sigma factor AlgU regulatory protein MucB	-5.32
PSYTB_24889	MBL fold metallo-hydrolase	-5.33
PSYTB_15445	flagellar hook protein FlgK	-5.34
PSYTB_13666	AI-2E family transporter	-5.35
PSYTB_14990	4-alpha-glucanotransferase	-5.35
PSYTB_27092	alpha/beta hydrolase	-5.37
PSYTB_15395	flagellar biosynthesis protein FlgB	-5.37
PSYTB_25731	—	-5.40
PSYTB_14745	hypothetical protein	-5.45
PSYTB_21360	type VI secretion protein	-5.51
PSYTB_32112	—	-5.51
PSYTB_15410	flagellar hook protein FlgE	-5.56
PSYTB_15705	coproporphyrinogen III oxidase	-5.58
PSYTB_09286	methylmalonate-semialdehyde dehydrogenase	-5.68
PSYTB_24879	pyridine nucleotide-disulfide oxidoreductase	-5.68
PSYTB_15575	flagellar basal body-associated protein FliL	-5.69
PSYTB_09281	omega amino acid--pyruvate aminotransferase	-5.72
PSYTB_25946	hypothetical protein	-5.80

Gene locus	Predicted function	Fold change (Wild type/Δ*psyI* mutant)
PSYTB_15400	flagellar basal body rod protein FlgC	-5.83
PSYTB_18809	amine oxidase	-5.85
PSYTB_04645	glycosyl transferase	-5.93
PSYTB_00579	trehalose synthase	-5.94
PSYTB_02589	gamma-glutamylputrescine oxidoreductase	-5.99
PSYTB_17235	ubiquinol oxidase subunit II, cyanideinsensitive	-6.01
PSYTB_27842	sorbosone dehydrogenase	-6.02
PSYTB_25489	hypothetical protein	-6.03
PSYTB_19281	glutamine amidotransferase	-6.09
PSYTB_27492	pyruvate dehydrogenase	-6.11
PSYTB_16510	cardiolipin synthase B	-6.13
PSYTB_16600	hypothetical protein	-6.18
PSYTB_05120	Fe-S oxidoreductase	-6.20
PSYTB_20051	hypothetical protein	-6.21
PSYTB_14228	hypothetical protein	-6.24
PSYTB_22435	serine dehydratase	-6.24
PSYTB_10558	DEAD/DEAH box helicase	-6.32
PSYTB_15405	flagellar basal body rod modification proteinFlgD	-6.33
PSYTB_09761	alcohol dehydrogenase	-6.33
PSYTB_15365	flagellar basal body P-ring biosynthesis proteinFlgA	-6.34
PSYTB_02324	GNAT family acetyltransferase	-6.34
PSYTB_03576	serine kinase/phosphatase	-6.41
PSYTB_00455	DNA topoisomerase	-6.46
PSYTB_07071	hypothetical protein	-6.56
PSYTB_00804	hypothetical protein	-6.68
PSYTB_16670	DNA repair protein	-6.69
PSYTB_11423	hypothetical protein	-6.72
PSYTB_17870	phospholipid-binding protein	-6.72
PSYTB_15320	3-oxoacyl-ACP synthase	-6.79
PSYTB_27167	hypothetical protein	-6.82
PSYTB_09681	hypothetical protein	-6.87
PSYTB_03144	chemotaxis protein	-6.97
PSYTB_16300	hypothetical protein	-7.08
PSYTB_09766	acetyltransferase	-7.14
PSYTB_10026	MFS transporter	-7.17
PSYTB_07076	hypothetical protein	-7.19
PSYTB_11593	peptidase M42	-7.30
PSYTB_09771	pectin lyase	-7.30
PSYTB_03139	—	-7.37
PSYTB_18814	carbon-nitrogen hydrolase	-7.42
PSYTB_03751	antitoxin	-7.47
PSYTB_15540	flagellar assembly protein FliH	-7.49

Gene locus	Predicted function	Fold change (Wild type/Δ*psyI* mutant)
PSYTB_27132	hypothetical protein	-7.51
PSYTB_15615	flagellar biosynthesis protein FlhA	-7.51
PSYTB_10088	hypothetical protein	-7.56
PSYTB_11563	membrane protein	-7.59
PSYTB_03741	hypothetical protein	-7.61
PSYTB_27552	hypothetical protein	-7.65
PSYTB_15595	flagellar biosynthesis protein FliP	-7.68
PSYTB_23091	bacterioferritin	-7.70
PSYTB_15315	transferase	-7.74
PSYTB_11598	GNAT family acetyltransferase	-7.77
PSYTB_09401	amino acid transporter LysE	-7.94
PSYTB_27232	hypothetical protein	-8.06
PSYTB_05085	hypothetical protein	-8.10
PSYTB_13090	—	-8.13
PSYTB_13095	hypothetical protein	-8.18
PSYTB_15310	oxidoreductase	-8.23
PSYTB_15335	aminotransferase	-8.23
PSYTB_07066	hypothetical protein	-8.25
PSYTB_27847	membrane protein	-8.27
PSYTB_32122	—	-8.33
PSYTB_15520	flagellar hook-basal body protein FliE	-8.37
PSYTB_14233	hypothetical protein	-8.38
PSYTB_15590	flagellar assembly protein FliO	-8.39
PSYTB_15305	hypothetical protein	-8.57
PSYTB_15600	flagellar biosynthetic protein FliQ	-8.81
PSYTB_11603	asparagine synthetase B	-8.88
PSYTB_05055	chemotaxis protein	-9.00
PSYTB_13546	hypothetical protein	-9.09
PSYTB_15620	flagellar biosynthesis regulator FlhF	-9.15
PSYTB_15420	flagellar basal body rod protein FlgF	-9.29
PSYTB_15325	glycosyl transferase	-9.43
PSYTB_15625	cobyrinic acid a,c-diamide synthase	-9.50
PSYTB_14123	MFS transporter	-9.61
PSYTB_23806	hypothetical protein	-9.67
PSYTB_15535	flagellar motor switch protein FliG	-9.86
PSYTB_15585	flagellar motor switch protein FliN	-9.88
PSYTB_15580	flagellar motor switch protein FliM	-9.96
PSYTB_15525	flagellar M-ring protein FliF	-10.06
PSYTB_27487	chemotaxis protein	-10.16
PSYTB_15425	flagellar basal body rod protein FlgG	-10.23
PSYTB_07166	hydroperoxidase II	-10.44
PSYTB_15430	flagellar L-ring protein	-10.56

Gene locus	Predicted function	Fold change (Wild type/Δ*psyI* mutant)
PSYTB_15510	sensor histidine kinase	-10.68
PSYTB_17240	hypothetical protein	-10.88
PSYTB_27087	hypothetical protein	-11.08
PSYTB_15440	flagellar rod assembly protein FlgJ	-11.26
PSYTB_15435	flagellar P-ring protein	-11.43
PSYTB_02674	aldehyde dehydrogenase	-11.71
PSYTB_15330	SAM-dependent methyltransferase	-11.94
PSYTB_14118	RND transporter	-12.40
PSYTB_03746	—	-12.59
PSYTB_15515	sigma-54-dependent Fis family transcriptionalregulator	-13.66
PSYTB_22130	topoisomerase II	-14.05
PSYTB_14113	hemolysin D	-14.79
PSYTB_00954	hypothetical protein	-84.09
